# MFSD-YOLO: A multi-scale feature detection network for pediatric wrist abnormalities in radiographic images

**DOI:** 10.1371/journal.pone.0340408

**Published:** 2026-02-05

**Authors:** Min Li, Yinping Jiang, Tao Xu, Peiyong Ji, Jingqi Hu, Xuejian Li, Wenlong Liu, Ruiqiang Guo

**Affiliations:** 1 Keimyung Academy at Changchun University, Changchun, China; 2 College of Electronic and Information Engineering, Changchun University, Changchun, China; 3 College of Mechanical and Vehicular Engineering, Changchun University, Changchun, China; Macau University of Science and Technology, MACAO

## Abstract

Timely and accurate diagnosis of wrist abnormalities, especially distal radius and ulna fractures, is critical in children and adolescents, yet complicated by skeletal immaturity, overlapping anatomy, and low-contrast fracture lines. To address these challenges, we propose MFSD-YOLO, a multi-scale detection model for pediatric wrist abnormality analysis. The model integrates a Cross-Stage Partial Progressive Multi-Scale Feature Aggregation (CSP_PMSFA) module inspired by GhostNet that applies lightweight multi-scale convolutions on partial channels with partial convolution and residual connections to reduce redundancy and enhance shallow texture and subtle fracture sensitivity. The Feature Pyramid Shared Convolution (FPSConv) module replaces pooling with shared dilated convolutions to expand the receptive field and capture multi-scale context without added cost. The C2 Bi-Level Routing Attention (C2BRA) module, based on C2PSA, uses regional routing and local enhancement to refine focus on relevant areas while balancing accuracy and speed. The Recursive Gradient Dynamic Feature Pyramid Network (RepGDFPN) optimizes top-down and bottom-up multi-scale fusion, reducing semantic loss and improving robustness. Finally, the Sliding Weight Adaptive Loss (SlideLoss) addresses class imbalance, enhancing detection of rare targets. Evaluated on the GRAZPEDWRI-DX dataset, MFSD-YOLO achieves 69.7% mAP@0.5, representing a 5.3% improvement over the baseline YOLOv11, while maintaining 10.8M parameters and 3.2 ms inference speed. These results validate the model’s effectiveness and its potential for real-world deployment in clinical pediatric radiographic analysis.

## 1. Introduction

Wrist abnormalities, encompassing both traumatic and non-traumatic conditions, are commonly observed in pediatric, adolescent, and young adult populations, and can substantially affect daily functional capacity as well as long-term musculoskeletal health [1,2]. Among these abnormalities, wrist fractures—including distal radius and distal ulna fractures—are the most prevalent, accounting for over 75% of all wrist fracture cases [3,4]. Epidemiological evidence indicates that the incidence of wrist abnormalities peaks during the pubertal growth spurt, a period characterized by rapid skeletal development and increased physical activity exposure, which elevates the risk of traumatic injury. Delayed or inaccurate diagnosis and intervention, particularly for lesions involving the physeal plate, may result in physeal arrest, malunion, or chronic functional impairment, ultimately exerting long-term effects on skeletal development and quality of life.

In clinical practice, X-ray radiography remains the first-line imaging modality for evaluating pediatric wrist abnormalities due to its low radiation dose, cost-effectiveness, and wide availability. Standard imaging protocols typically include posteroanterior (PA) and lateral views, which allow assessment of cortical integrity, fracture morphology, and bony alignment of major wrist structures, including the radius, ulna, and carpal bones. However, interpretation of pediatric wrist radiographs is considerably more challenging than in adults. Developing carpal bones at different growth stages exhibit significant variability in morphology, bone density, and grayscale distribution. Subtle fractures and physeal plates present similar grayscale characteristics, while overlying soft tissues further reduce local contrast. The combination of these multi-scale anatomical structures and low-contrast features renders pediatric wrist images inherently ambiguous and heterogeneous, thereby increasing the uncertainty of manual diagnosis. Clinical studies have reported misdiagnosis rates of 15%–26% in pediatric emergency departments and primary care settings, primarily attributed to reader fatigue, insufficient experience, or limited image quality [5–7].

With the rapid advancement of artificial intelligence (AI) and computer vision technologies, computer-aided diagnosis (CAD) systems have emerged as a crucial approach in medical image analysis [8,9]. Deep learning-based detection models can automatically learn underlying feature patterns from medical images, enabling an end-to-end analysis pipeline from image input to abnormality detection. Current mainstream object detection algorithms are broadly classified into two categories: two-stage and one-stage detectors. Two-stage methods, exemplified by the R-CNN series, achieve precise detection through sequential candidate region generation, classification, and regression [10–12]. One-stage methods, including the YOLO and SSD series, integrate localization and classification within a single framework, thereby balancing detection speed and accuracy [13–17]. In recent years, the incorporation of multi-scale feature fusion and attention mechanisms has further enhanced these models’ representational capacity for complex anatomical structures, resulting in superior performance in multi-scale object detection and real-time clinical tasks.

Despite significant progress in CAD systems across various medical imaging applications, research focusing specifically on pediatric wrist radiographs remains limited. Existing detection frameworks are generally designed for generic tasks and do not fully account for the anatomical characteristics and imaging patterns of developing bones. As a result, they often exhibit suboptimal performance in scenarios with low contrast, subtle textures, or pronounced scale variations. To address these limitations, this study proposes an anatomy-driven multi-scale feature detection network, named MFSD-YOLO. The model integrates imaging features with anatomical prior knowledge and employs a detection strategy centered on shallow texture enhancement, scale-adaptive feature fusion, and regional feature focusing. This approach aims to improve the model’s ability to identify small-scale, low-contrast abnormalities, such as early periosteal reactions and non-displaced fractures, thereby providing a reliable tool for early diagnosis and clinical decision support in pediatric wrist abnormalities.

The structure of this paper is organized as follows: Section 2 mainly reviews deep learning–based medical image detection methods and their applications in wrist abnormality recognition; Section 3 describes the proposed MFSD‑YOLO network architecture, the utilized dataset, and preprocessing workflow; Section 4 presents the experimental design, evaluation metrics, and comparative results, along with an analysis of the model’s detection performance and robustness; Section 5 discusses the model’s advantages, potential limitations, future research directions, and clinical deployment considerations; finally, Section 6 concludes with a summary of the key contributions and findings.

## 2. Related work

### 2.1. Pediatric wrist abnormality detection

Several recent studies have applied YOLO-based frameworks to wrist abnormality detection. Ju et al. established a YOLOv8 baseline on the GRAZPED-WRI-DX dataset, achieving a mean average precision (mAP) at IoU threshold 0.5 (mAP@0.5) of 63.8% [18]. Ahmed et al. proposed a dual-label assignment strategy in YOLOv10, improving performance in anatomically dense regions and achieving an accuracy of 51.9% [19]. To improve fine-grained feature discrimination, Ju et al. incorporated squeeze-and-excitation modules into YOLOv8, increasing mAP@0.5 to 67.07% [20]. Bouslimi et al. integrated SimAM attention and AC-BiFPN into YOLOv10, reporting 88.5% mAP@0.5 and 97.4% precision [21]. Tariq et al. devel-oped a ResNet-GAM model that combines residual learning and guided attention, achieving 79.9% accuracy and 63.9% recall [22]. Similarly, Chien et al. proposed YOLOv8-AM using multiple attention modules, which achieved 65.8% mAP@0.5 [23]. For resource-constrained environments, Ferdi et al. introduced a lightweight YOLOv11-based CAD system using GhostNet and C3Ghost modules to reduce parameter count and improve inference speed while preserving accuracy [24].

These studies collectively demonstrate the adaptability of YOLO-based frameworks for pediatric wrist abnormality detection and their potential as clinical assistive tools to enhance diagnostic efficiency and support radiologists in decision-making.

### 2.2. Advances in multi-scale feature fusion

Lesions in medical images exhibit pronounced multi-scale characteristics. Large-scale abnormalities often span substantial anatomical regions, such as entire bones or organ segments, covering hundreds or even thousands of pixels. These regions provide critical contextual and morphological information that aids in assessing the extent and anatomical positioning of the lesion. In contrast, small-scale abnormalities occupy only localized areas consisting of a few dozen pixels, manifesting as subtle nodules, fine fracture lines, or microhemorrhages. These regions contain essential texture and boundary cues that are indispensable for detecting early-stage or fine-structural pathologies. Consequently, single-scale feature extraction is often insufficient to balance global contextual understanding with local detail preservation, leading to missed small lesions or incomplete semantic representation.

To address this challenge, multi-scale feature fusion (MSFF) integrates features across multiple layers and receptive fields, enabling simultaneous perception of fine-grained details at high resolutions and global structures at low resolutions. Lin et al. introduced the Feature Pyramid Network (FPN), which employs a top-down pyramid structure to merge features from different layers, establishing a foundational framework for subsequent multi-scale detection studies [25]. Building on this concept, Liu et al. developed a 3D FPN architecture for pulmonary nodule detection, which significantly improved recognition across nodules of varying sizes [26]. Sun et al. incorporated an enhanced FPN into a U-Net framework for brain tumor segmentation, achieving superior segmentation precision [27]. Similarly, Zhang et al. proposed a 3D FPN-based single-stage lung nodule detection network, which effectively balanced detection performance across both large and small nodules [28]. Furthermore, Xu et al. designed an Enhanced FPN (EFPN) module tailored to the unique characteristics of medical images, improving the adaptability of multi-scale feature fusion to medical imaging contexts [29].

In summary, multi-scale feature fusion enables the joint capture of global structures and fine local details, providing a robust mechanism to handle the diverse texture scales, grayscale ambiguities, and anatomical overlaps often present in pediatric wrist radiographs.

### 2.3. YOLOv11 overview

**Fig 1:**
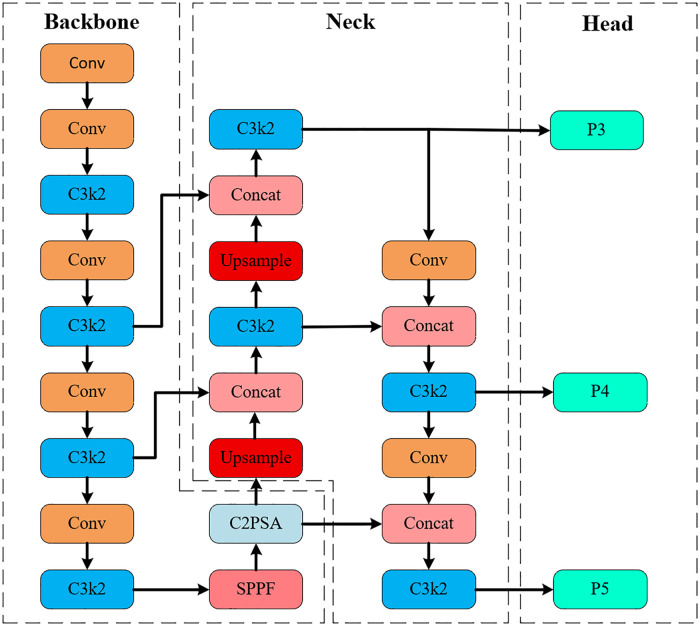
The overall structure of YOLOv11.

YOLOv11, released by Ultralytics in 2024, represents a new generation of real-time object detection models. It retains the hallmark efficiency of single-stage detectors while introducing several key architectural improvements to boost detection performance. As illustrated in Fig 1, the network comprises three main components: the backbone, neck, and detection head. In the backbone, the conventional C2f blocks used in YOLOv8 are replaced with more lightweight C3k2 modules, enabling better feature representation with reduced computational redundancy. The neck integrates an improved Spatial Pyramid Pooling-Fast (SPPF) module for receptive field expansion, followed by the Cross-Stage Partial Spatial Attention (C2PSA) module to enhance spatial awareness through attention-based refinement. In the detection head, an anchor-free, decoupled structure is adopted. The classification branch utilizes depthwise separable convolutions to lower computational cost, whereas the regression branch retains standard convolutions to maintain localization precision.

## 3. Materials and methods

### 3.1. GRAZPEDWRI-DX dataset

This study utilized the publicly available GRAZPEDWRI-DX dataset, jointly established by the Medical University of Graz and the University Hospital Graz (Austria) [30]. The dataset includes 6,091 unique pediatric patients (mean age: 10.9 years; range: 0.2–19 years; 2,688 females, 3,402 males, 1 unknown), comprising 10,643 radiographic examinations and 20,327 wrist radiographs collected between 2008 and 2018. All radiographs were obtained from routine clinical procedures performed at the Department of Radiology, University Hospital Graz, covering multiple anatomical regions such as the radius, ulna, and carpal bones, with various projection angles including posteroanterior (PA), lateral (LAT), and oblique (OBL) views.

All images were stored in de-identified DICOM format, and personally identifiable information (such as patient name, date of birth, and study ID) was automatically removed using the built-in anonymization module of the Hospital Information System (HIS).

**Fig 2:**
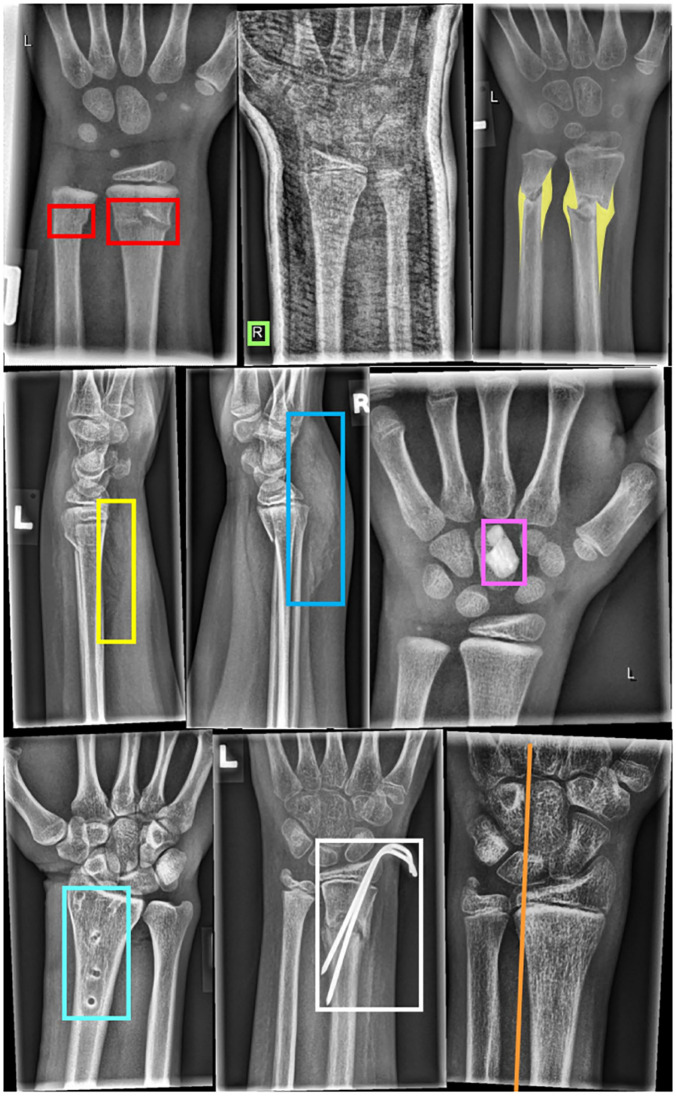
Examples of different objects labeled by human experts. From left to right in the first row are: Fracture, Text, Periosteal reaction. From left to right in the second row are: Pronator quadratus sign, Soft tissue swelling, Foreign body. From left to right in the last row are: Bone abnormality, Metal, and Axis. The X-ray in the middle of the first row shows a plaster cast. The images on the far right of the first and third rows are labeled as osteopenia.

The dataset contains nine object categories: periosteal reaction, fracture, metal, pronator sign, soft tissue, bone anomaly, bone lesion, foreign body, and text. Among these,the “text” label represents embedded annotations in the image (e.g., projection orientation or examination information), which are visible in most X-rays. Boneanomaly constitutes a heterogeneous group representing various osseous abnormalities, ranging from drill holes to Madelung deformities, while bonelesion refers to bone neoplasms such as osteomas. Excluding the “text” category, fracture is the most prevalent abnormality, followed by periosteal reaction and metal structures, whereas boneanomaly, bone lesion, and foreignbody occur less frequently. Notably, the fracture category in GRAZPEDWRI-DX represents wrist-level fractures as a unified class without further specifying anatomical sites (e.g., distal radius or ulna). Therefore, all “fracture” results reported in this study refer to wrist-level fracture abnormalities rather than site-specific lesions. The category distribution is summarized in Table 1, and representative examples from the training subset are shown in Fig 2.

**Table 1:** Class distribution.

Abnormality	Instances	Ratio
**Boneanomaly**	192	0.94%
**Bonelesion**	42	0.21%
**Foreignbody**	8	0.04%
**Fracture**	13550	66.6%
**Metal**	708	3.48%
**Periosteal reaction**	2235	11.0%
**Pronatorsign**	566	2.78%
**Softtissue**	439	2.16%
**Text**	20274	99.74%

### 3.2. Annotation and quality control

Image annotation was conducted on the Supervisely online platform (Deep Systems LLC, Moscow, Russia). Three certified pediatric radiologists (S.T., E.N., and E.S.), each with 6–29 years of experience in musculoskeletal imaging, were responsible for verifying the accuracy and consistency of all annotations. The annotation database was hosted on a secure server, enabling remote collaboration via a web interface. In addition to the three primary reviewers, local radiologists, visiting scholars, and medical students participated in the initial labeling process. The annotation work was carried out over a period spanning March 2018 to February 2022.

Annotations were performed using bounding boxes, polylines, and polygons to precisely delineate fracture lines, metallic implants, and abnormal structures. To evaluate inter-observer agreement, the Intersection over Union (IoU) metric was employed, yielding an average IoU of 0.70 (P1 = 0.22, P25 = 0.63, P50 = 0.73, P75 = 0.80, P99 = 0.94), which demonstrates a high degree of consistency among radiologists.

A comprehensive data quality control procedure was applied to ensure dataset integrity, combining both automated and manual inspections. This procedure included verification of the consistency between DICOM headers and annotation files, inspection of image resolution, exposure parameters, and completeness of annotations, exclusion of overexposed, blurred, or duplicate images, and confirmation that all images were fully anonymized. These measures collectively ensured that the dataset maintains high reliability with respect to anonymization, annotation accuracy, and structural consistency.

### 3.3. Ethical statement

The establishment and release of the GRAZPEDWRI-DX dataset underwent rigorous ethical review and was approved by the Ethics Committee of the Medical University of Graz (Approval No. EK 31–108 ex 18/19). All procedures fully complied with the Declaration of Helsinki regarding ethical principles for medical research. The dataset was classified as a retrospective anonymized imaging study, and thus informed consent was waived.

All images were collected from routine clinical examinations. During data export, the HIS system automatically anonymized all DICOM files by removing personally identifiable information such as patient names, dates of birth, and examination numbers, ensuring that individual identities could not be traced. Data storage and access followed the data security policies of the University Hospital Graz, and all files were manually and automatically verified before being publicly released. The dataset is currently available on the Figshare platform (https://figshare.com/articles/dataset/GRAZPEDWRI-DX/14825193) for academic and non-commercial use only. This study did not involve direct access to raw patient data or identifiers; all experiments were conducted exclusively on anonymized images, and therefore no additional ethical approval or patient consent was required.

### 3.4. Model overview and key contributions

**Fig 3:**
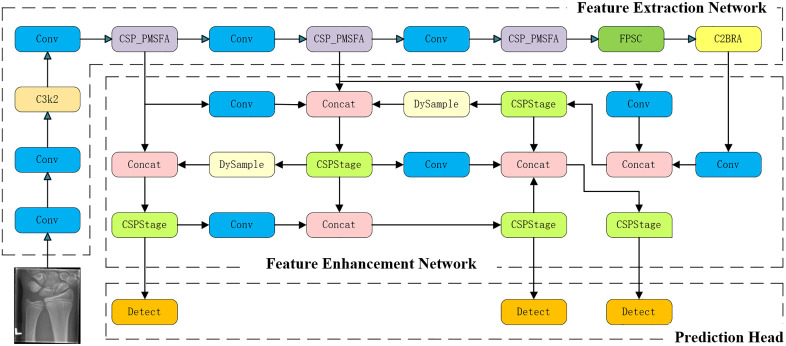
The overall structure of MFSD-YOLO.

This study proposes a multi-scale detection framework for pediatric wrist imaging, termed MFSD-YOLO, as illustrated in Fig 3. The proposed framework comprises three key stages: feature extraction, feature enhancement, and prediction. The methodological innovations of this study are primarily reflected in the design and integration of five core modules:

1. **CSP_PMSFA:** To address the challenges of low contrast between cortical and trabecular bone, as well as the weak early periosteal reaction signals in pediatric wrist radiographs, the CSP_PMSFA module incorporates progressive multi-scale convolution and partial convolutional branches into the CSP backbone. This enables the network to capture fine-grained texture at shallow layers while maintaining stable structural semantics at deeper layers. Through cross-scale feature fusion and residual guidance, CSP_PMSFA enhances sensitivity to minor cortical disruptions while preserving bone continuity, thereby providing stable and structure-aware representations for subsequent feature learning.
2. **FPSConv:** Considering the substantial scale differences and semantic discontinuities among the radius, ulna, and carpal bones, FPSConv employs shared convolutional kernels to achieve semantic alignment across multiple feature maps, complemented by dilated convolutions with varying rates for multi-scale context modeling. This mechanism corresponds to the anatomical hierarchy of the wrist—shared kernels maintain spatial consistency, while dilation enlarges the receptive field to capture adjacent bony structures. By establishing semantic bridges between global skeletal frameworks and local bone elements, FPSConv effectively improves adaptability to cross-scale variations, enabling unified interpretation of both macroscopic morphology and subtle abnormalities.
3. **C2BRA:** In pediatric wrist radiographs, crucial diagnostic cues often reside at cortical boundaries or soft tissue transition zones, where weak signals and high noise frequently obscure meaningful features. The C2BRA module introduces a bidirectional residual attention mechanism that jointly leverages channel reweighting and spatial focusing to enhance semantic representation. The forward path captures global anatomical context, whereas the backward residual pathway reinforces local structural discontinuities, thereby improving the network’s ability to focus on blurred cortical edges and subtle fracture lines.
4. **RepGDFPN:** The complex and overlapping multi-layer bony structures of pediatric wrists often lead to semantic drift and structural discontinuity in conventional feature pyramid fusion. To mitigate this, the RepGDFPN module introduces a recursive gradient modulation mechanism into the traditional FPN framework, dynamically correcting inter-scale discrepancies through iterative feedback. This recursive optimization significantly enhances structural integrity and boundary clarity during multi-scale feature fusion, producing anatomically consistent feature representations for final detection.
5. **SlideLoss:** To handle severe class imbalance within the dataset, SlideLoss applies a sliding weighting strategy that dynamically adjusts loss weights according to sample difficulty and prediction confidence during training. This adaptive loss design directs gradient optimization toward challenging or infrequent lesion samples, improving detection sensitivity and robustness in low-contrast and atypical cases.

In summary, this study integrates algorithmic design with pediatric wrist anatomical priors, establishing a coherent mapping from structural hierarchy to functional feature focus across feature extraction, semantic fusion, and loss optimization.

### 3.5. CSP_PMSFA module

Pediatric wrist radiographs exhibit pronounced structural heterogeneity. Due to incompletely ossified centers, the grayscale contrast between cortical and trabecular bone is low, and uneven development across different skeletal units often results in texture discontinuities, blurred density transitions, and interrupted boundary signals. These imaging characteristics make it challenging for models to accurately capture fine-grained structures across hierarchical levels in the distal radial metaphysis and epiphyseal plate regions, particularly when distinguishing physiological growth plates from subtle fracture lines, where feature confusion frequently occurs. The original YOLOv11 backbone employs the C3k2 block, a CSP-based bottleneck derived from C2f that applies two sequential convolutions to all channels within each residual branch. While effective in maintaining gradient flow, its uniform kernel configuration and equal processing of all channels result in limited receptive-field diversity and computational redundancy, reducing sensitivity to subtle fracture boundaries in pediatric wrist X-rays.

**Fig 4:**
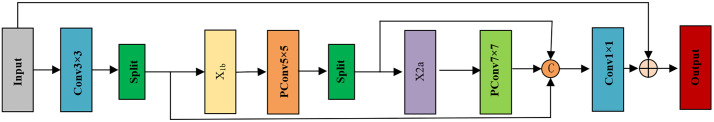
The overall architecture of the CSP_PMSFA module.

To address these issues, this study proposes CSP_PMSFA as a replacement for the original C3k2 structure. Within the CSP backbone, CSP_PMSFA introduces progressive multi-scale convolutional branches [31] and a partial convolution strategy [32], which expand the receptive field hierarchically and selectively extract features across channels, enabling a continuous mapping from global skeletal morphology to local fissure textures. As shown in Fig 4, CSP_PMSFA applies standard convolutions to only a subset of channels, generating partially redundant features through low-cost operations to reduce computational overhead. Partial Convolution (PConv) is incorporated along the gradient propagation path, extracting multi-scale features layer by layer using grouped 5 × 5 and 7 × 7 convolutions, and encoding contextual structural information from different sub-channels.

**Fig 5:**
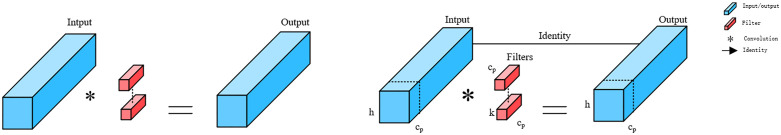
Comparison between standard convolution and partial convolution.


The working principles of Standard Convolution and Partial Convolution are illustrated in Fig 5, compared with standard convolution, PConv selectively computes a subset of the feature channels. Its computational cost can be approximated as:

$$\begin{array}{c} {FLOP}_{S_{PConv}}=h\times w\times k^{\text{2}}\times c_{p}^{\text{2}} \end{array}$$ (1)

where h and w are the spatial dimensions of the feature map, k is the kernel size, and $c_{p}$ denotes the number of selected participating channels. When the channel ratio r = 0.25, PConv requires only about one-fourth of the FLOPs and memory accesses compared to a full standard convolution.

The CSP_PMSFA module adopts a progressive multi-scale design based on this principle. Specifically, the input feature map $X\in R^{C\times H\times W}$ is first processed by a 3 × 3 convolutional layer, then split into $X_{\text{1}a}\text{and}\;X_{\text{1b}}\;$.${\;X}_{\text{1}a}$ undergoes grouped 5 × 5 convolution to produce an intermediate feature map $X_{\text{2}\;}$,Which is further split into $X_{\text{2}a}$ and $X_{\text{2}b}$. The former is then processed by a grouped 7 × 7 convolution to generate a high-level semantic feature map $X_{\text{3}}$. The final representation is:


$$\begin{array}{c} Y=Concat\left(X_{\text{3}},X_{\text{2}b},X_{\text{1}b}\right)\in R^{C\times H\times W} \end{array}$$ (2)

$$\begin{array}{c} Z={Conv}_{\text{1}\times \text{1}}(Y)+X \end{array}$$ (3)

The total computational cost of the CSP_PMSFA module can be approximated as:


$$\begin{array}{c} {FLOP}_{S_{CSP\_PMSFA}}\approx H\cdot W\cdot \left[C+rC\cdot k_{\text{1}}^{\text{2}}+\frac{rC}{\text{2}}\right]\cdot k_{\text{2}}^{\text{2}}+\frac{rC}{\text{4}}\cdot k_{\text{3}}^{\text{2}} \end{array}$$ (4)

Where r denotes the proportion of channels involved in multi-scale computation (typically set to 0.5), while $k_{\text{1}}=\text{3},k_{\text{2}}=\text{5},k_{\text{3}}=\text{7}\;$represent the kernel sizes used in the respective convolutional branches.

In summary, CSP_PMSFA establishes a “macro-to-micro” modeling pathway that aligns feature hierarchies with the anatomical structure of the pediatric wrist. Low-level branches focus on global skeletal morphology and cortical continuity, while high-level branches target high-frequency abnormal regions such as the epiphyseal plate and fracture fissures, thereby achieving high sensitivity for detecting subtle fractures in immature bones.

### 3.6. FPSConv module

Pediatric wrist radiographs exhibit pronounced spatial hierarchical characteristics. The distal radius and ulna represent large-scale, long-bone structures, whereas the carpal bones, metacarpal bases, and joint spaces exhibit small-scale, densely packed morphologies. Within a single radiograph, the grayscale distribution and boundary textures of bone tissues across these hierarchical levels vary nonlinearly, which may cause semantic drift during cross-scale feature extraction. Traditional feature extraction modules, such as SPPF, rely on discrete pooling operations to expand the receptive field during multi-scale feature aggregation. Although this strategy integrates information across layers, it disrupts spatial continuity and results in the loss of fine-grained textures. For pediatric wrist radiographs, where the detection of subtle fissures or low-contrast boundary regions is crucial, such limitations reduce the model’s capacity for accurate structural identification.

To address this, we propose the FPSConv module, which replaces conventional multi-branch pooling with kernel-sharing atrous convolutions [33,34]. This design achieves continuous multi-scale feature representation and consistent contextual modeling while maintaining computational efficiency.

**Fig 6:**
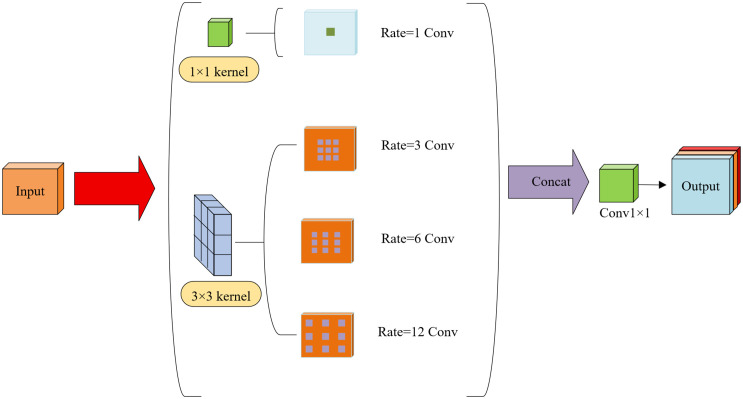
The overall architecture of the FPSConv module.


As illustrated in Fig 6, the input feature map is first compressed from Cin channels to Cin/2 channels via a 1 × 1 convolution to reduce computational cost. Then, the same 3 × 3 convolution kernel $W_{shared}$ is applied with different dilation rates d∈{3,6,12} through atrous convolutions, formulated as:

$$\begin{array}{c} g(p)=\sum_{s+d\cdot t=p}f(s)\times k(t) \end{array}$$ (5)

Where g(p) is the output feature at position p,f(s) is the input feature at location s, k represents the convolutional kernel weights, and d is the dilation rate. When d > 1, zeros are inserted between kernel elements to enlarge the receptive field without increasing the number of parameters. Because all branches share the same kernel weights, features at different scales jointly contribute to weight updates, enabling the model to simultaneously capture epiphyseal plate details (small dilation) and global contours of the distal radius and ulna (large dilation).This process is formalized as:


$$\begin{array}{c} Y_{d}=Conv\left(X,W_{shared},dilation=d\right) \end{array}$$ (6)

where X is the compressed input feature map and Y_d_ denotes the output of each branch with different dilation rates. Finally, outputs from all branches are concatenated along the channel dimension and fused via a 1 × 1 convolution to produce the final feature map:


$$\begin{array}{c} Y_{out}=Conv\text{1}\times \text{1}\left(Concat\left(\{Y_{d\text{1}},Y_{d\text{2}},\;Y_{d\text{3}}\}\;\right)\right)+X \end{array}$$ (7)

This architecture achieves continuous propagation of multi-scale features and efficient weight sharing, enhancing feature integrity and spatial consistency while keeping computation compact. Shared kernels ensure anatomically consistent responses across different receptive fields, meaning that the same bone region maintains consistent texture representation across multi-scale feature maps. The multi-dilation mechanism dynamically adjusts the “observation focus”: small dilation rates concentrate on local structures such as epiphyseal plates and fissures, whereas large dilation rates integrate global bone frameworks and soft tissue context. Through this collaborative design, FPSConv balances global semantic awareness with fine-detail recognition, exhibiting improved robustness and medical interpretability.

### 3.7. C2BRA module

Pediatric wrist radiographs exhibit critical diagnostic regions concentrated at the epiphyseal plates and periosteal reaction zones, characterized by subtle grayscale variations, discontinuous boundaries, and overlapping soft tissue signals. Conventional convolutional structures often average local anomalous features in these regions, obscuring subtle cortical interruptions and early bone lesions.Traditional self-attention mechanisms possess strong global modeling capabilities for high-resolution medical images; however, their computational complexity grows quadratically with the spatial resolution of the feature maps. This results in low inference efficiency, making it challenging to meet the clinical demands for real-time processing and detailed feature capture. To address this issue, we propose the C2BRA module based on the Bi-Level Routing Attention (BRA) mechanism, built upon the C2PSA module in YOLOv11 [35]. The design aims to significantly reduce computational overhead while maintaining high representational power, thereby adapting to the detection requirements of high-resolution medical imaging.

**Fig 7:**
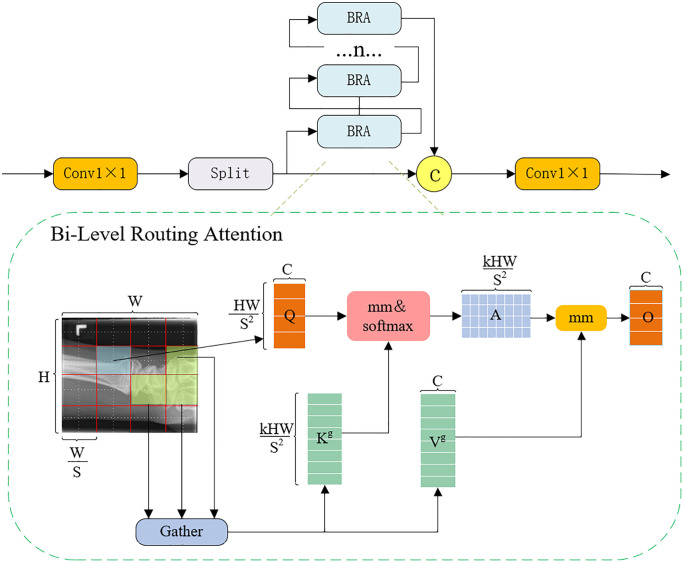
The overall architecture of the C2BRA Module.

As illustrated in Fig 7, C2BRA employs a dual-path architecture: a regional routing path and a local enhancement path. The regional routing path constructs a sparse semantic routing graph via regional partitioning and semantic similarity, selectively emphasizing diagnostically critical anatomical regions such as the distal radial metaphysis and epiphyseal plates. This reduces unnecessary computation and reinforces semantic consistency across scales. The local enhancement path applies lightweight depthwise separable convolutions to the Value tensor [36], restoring fine-grained features attenuated by sparsification and enhancing the model’s sensitivity to cortical discontinuities, subtle fracture lines, and periosteal reaction zones.

Specifically, given an input feature map $X\in R^{B\times C\times W}$, the C2BRA module first applies a set of 1 × 1 convolutional projections to generate the query (Q), key (K), and value (V) vectors:


$$\begin{array}{c} \left(Q,K,V)={Conv}_{\text{1}\times \text{1}}(X\right) \end{array}$$ (8)

The feature map is then evenly divided into $n_{Win}\times n_{Win}$ spatial regions. Within each region, average pooling is applied to the query and key tensors to derive the region-level representations, $Q_{r}=AvgPool(Qand\;K_{r}=AvgPool(K)$, respectively. The region-wise similarity matrix is defined as:


$$\begin{array}{c} A_{r}=Q_{r}^{T}K_{r} \end{array}$$ (9)

where each element represents the semantic relevance between two regional features. To enforce sparsity, only the top-kmost relevant regions are retained for each query, forming a region routing graph $G_{r}$, with the associated index set defined as:


$$\begin{array}{c} I_{r}=TopK\left(A_{r}\right) \end{array}$$ (10)

Based on this routing map, the attention computation for each spatial query position $Q_{i}$ is limited to the key and value tokens located within its top-k target regions. The localized attention is formulated as:


$$\begin{array}{c} Attention\left(Q_{i},{\left\{K_{j}\right\}}_{j\in I_{r}(i)},{\left\{V_{j}\right\}}_{j\in I_{r}(i)}\right)=\sum_{j\in I_{r}(i)}Softmax\left(\frac{Q_{i}^{T}K_{j}}{\sqrt{d}}\right)V_{j} \end{array}$$ (11)

where d denotes the dimensionality of the query vectors. By restricting token interactions to only the most relevant regions, computational complexity reduces from $O(N^{\text{2}})$to O(kN), significantly enhancing inference efficiency on high-resolution feature maps.

The final output of the C2BRA module is defined as:


$$\begin{array}{c} O=Attention(Q,K,V)+DWConv(V) \end{array}$$ (12)

In summary, C2BRA efficiently integrates global semantic modeling with local structural refinement. By emphasizing key anatomical regions and restoring high-frequency microstructural details, the module ensures accurate representation of clinically relevant features in regions with weak radiographic contrast. This design achieves a dynamic balance between computational efficiency and high-fidelity feature representation, providing robust and interpretable high-resolution detection for complex pediatric wrist anatomy.

### 3.8. RepGDFPN module

Pediatric wrist radiographs exhibit pronounced spatial heterogeneity and overlapping anatomical structures. The distal radial metaphysis, carpal bones, and epiphyseal plates form multi-layered superimposed structures, where grayscale signals are continuous but textural boundaries are often blurred. Differences in ossification levels introduce semantic gaps between the high-level cortical bone and low-level trabecular bone, making it challenging for traditional top-down feature fusion structures to preserve structural continuity and boundary clarity. While the Path Aggregation Network (PAN) employed in the original YOLOv11 enhances bidirectional information flow, its fixed interpolation upsampling and conventional convolutional fusion can cause semantic drift in complex imaging scenarios, weakening small-scale structures such as epiphyseal lines and fissures.

**Fig 8:**
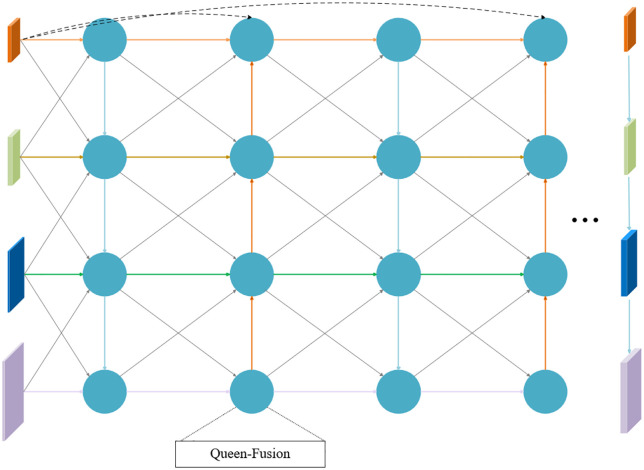
The structure diagram of the GFPN module.

**Fig 9:**
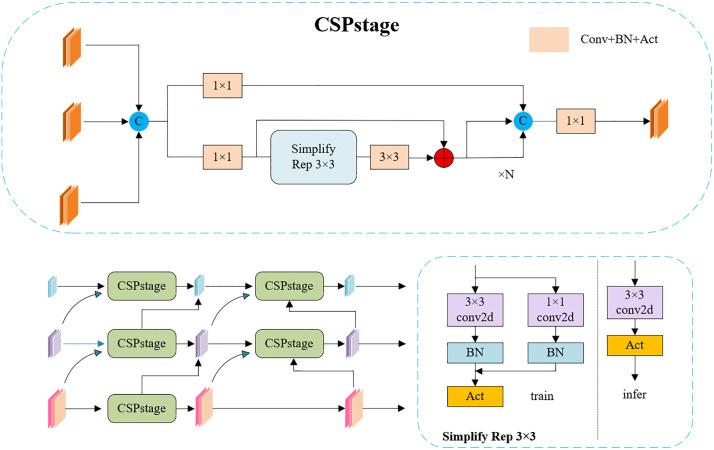
The overall architecture of the RepGFPN Module.

To address these limitations, a Generalized Feature Pyramid Network (GFPN) was proposed as an improved feature fusion structure (Fig 8). GFPN introduces bidirectional lateral and vertical connections across multi-scale layers, facilitating richer feature interaction and enhancing the integration of low-level spatial details with high-level semantic information. However, frequent up- and down-sampling operations increase inference latency and can induce semantic misalignment in small-scale bony structures [37,38]. Building upon GFPN, DAMO-YOLO further introduced the Efficient Reparameterized GFPN (RepGFPN) [39], which integrates the Efficient Layer Aggregation Network (ELAN) [40] structure with reparameterized convolution modules (CSPStage) to achieve scale-specific channel allocation, balancing computational cost with feature representation capability. The overall architecture of RepGFPN is illustrated in Fig 9. Conceptually, this structure can be interpreted as hierarchical modeling of pediatric wrist anatomy: shallow branches focus on local textures of cortical and trabecular bone, while high-level branches integrate global semantics of the carpal and joint structures, thereby providing more stable structural representation.

In RepGFPN, the feature map is first split into two parallel branches. In the upper branch, the channel dimension is reduced by a 1 × 1 convolution and the result is directly forwarded to the final concatenation. In the lower branch, an Efficient Layer Aggregation Network (ELAN) strategy is applied, consisting of alternating 1 × 1 and 3 × 3 convolution layers, where the 3 × 3 layers adopt a heavy re-parameterization mechanism. Outputs from different layers are preserved to enrich multi-scale representation. The features from both branches are concatenated and passed through a 1 × 1 convolution to generate the final fused feature map.

Within the ELAN pathway, the CSPStage module employs RepConv [41], a re-parameterized convolution block that fuses multiple branches into a single equivalent convolution during inference. This design reduces latency while retaining the representational capacity of multi-branch structures. By integrating ELAN with CSPStage, the fusion block achieves efficient multi-scale feature aggregation, enhancing both detection accuracy and inference speed.

Nevertheless, RepGFPN’s upsampling still relies on fixed interpolation, whose sampling positions cannot adapt to the feature distribution. This rigidity can introduce spatial distortion and semantic misalignment in high-frequency regions, such as cortical edges, epiphyseal plates, and joint gaps, adversely affecting the detection stability of small-scale structures. To overcome this issue, we propose the RepGDFPN, which retains the efficient architecture of RepGFPN while incorporating the DySample dynamic upsampling mechanism to achieve spatially and semantically adaptive alignment.

**Fig 10:**
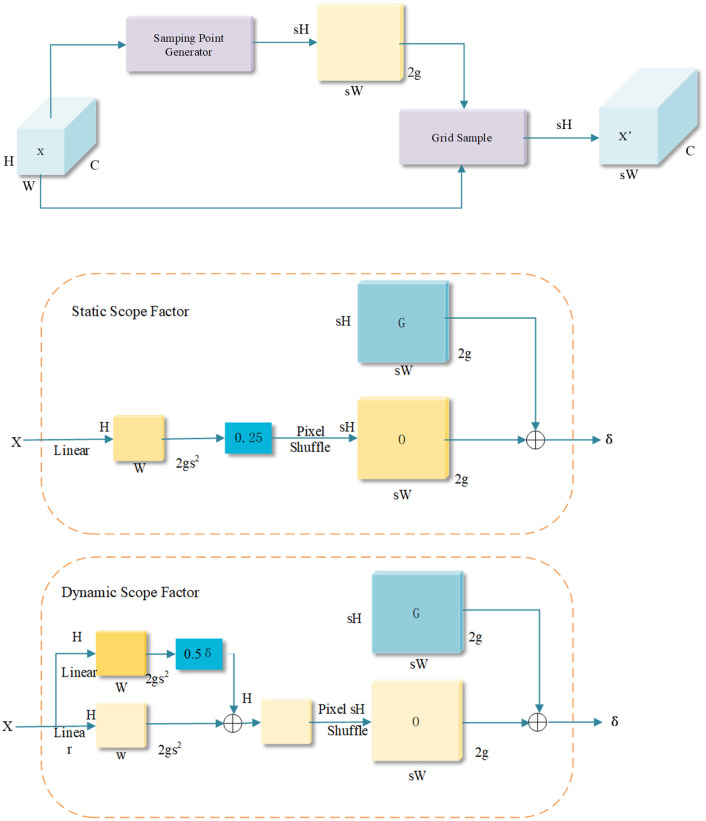
Sampling point generator in Dysample: (a) Sampling Based Dynamic UpSampling.(b) Sampling Point Generator in Dysample.

DySample mitigates the limitations of fixed interpolation by formulating upsampling as a learnable coordinate sampling process [42], enabling RepGDFPN to maintain spatial fidelity and grayscale consistency of cortical edges and joint gaps during multi-scale feature fusion. Combined with a lightweight recursive feature feedback path, RepGDFPN dynamically corrects semantic discrepancies across layers during fusion, ensuring anatomically consistent hierarchical propagation of features in pediatric wrist radiographs. This process is defined as:


$$\begin{array}{c} \hat{f}(x)=\sum_{k=\text{1}}^{K}w_{k}(x)\cdot f\left(x+\Delta_{k}(x)\right) \end{array}$$ (13)

where $\hat{f}(x)$ denotes the reconstructed feature at position x, f(· is the input feature map, $\Delta_{k}(x$ represents the spatial offset of the k-th sampling point, and $w_{k}(x)$ is the corresponding linear interpolation weight. Fig 10 illustrates the sampling-based dynamic upsampling and module design in DySample.

The effectiveness of the proposed sampling-driven dynamic upsampling strategy is illustrated in Fig 10 (a). Consider an input feature map X with dimensions $C\times H_{\text{1}}\times W_{\text{1}}$ and a coordinate sampling set δ of $\text{2}\times H_{\text{2}}\times W_{\text{2}}$, where the first channel stores the x and y offsets. The grid_sample operation is used to perform spatial resampling on the feature map X based on the coordinates defined in δ, utilizing bilinear interpolation to produce an upsampled output $X^{\prime }\in R^{C\times H_{\text{2}}\times W_{\text{2}}}$. This resampling can be expressed as:


$$\begin{array}{c} X^{\prime }=grid_{sample(X,\delta )} \end{array}$$ (14)

Assuming the upsampling ratio is s, and the input tensor X has shape  C×H×W, a linear transformation layer is applied, mapping C channels to 2$s^{\text{2}}$channels, to generate an offset tensor $O\in R^{\text{2}s^{\text{2}}\times H\times W}$. Through a pixel rearrangement process (as described in [43])this tensor is reshaped to a size of 2×sH×sW, aligning with the spatial resolution of the target output. The final sampling coordinates δ are then computed by combining the learned offset O with a predefined regular sampling grid G, as formulated below:


$$\begin{array}{c} O=Linear(X) \end{array}$$ (15)

$$\begin{array}{c} \delta =G+O \end{array}$$ (16)

Ultimately, the refined feature map X′ with resolution C×sH×sWis reconstructed through the sampling mechanism defined by δ and the grid_sample operation, as described in Equation (14).

### 3.9. SlideLoss loss function

The distribution of abnormality classes in pediatric wrist radiographs is highly imbalanced. Fracture samples account for approximately 66.6% of the dataset, whereas clinically important but subtler abnormalities—such as periosteal reactions, bone lesions, and foreign bodies—constitute only 0.2%–11% of samples. This long-tailed distribution causes the model to bias towards the dominant classes during training, often neglecting rare lesions with subtle grayscale variations and complex morphologies. Traditional loss functions, such as Cross-Entropy or Focal Loss, fail to adequately balance sample contributions, leading to the common issue of “overfitting easily classified samples while ignoring difficult-to-classify ones,” which limits sensitivity and stability in detecting small lesions.

To address this challenge, SlideLoss is adopted as the classification loss function in MFSD-YOLO. By implementing a dynamic weighting mechanism, SlideLoss balances the gradient contributions of samples with varying difficulty levels during training [44], thereby enhancing the model’s ability to accurately recognize rare and subtle pediatric wrist abnormalities.

SlideLoss is a weight-modulated loss function that dynamically adjusts sample weights based on the Intersection over Union (IoU) between predicted and ground truth boxes, emphasizing learning on “boundary samples”. Specifically, SlideLoss sets the average IoU of all positive samples as the classification threshold μ and assigns different difficulty levels and corresponding weights according to each prediction’s IoU x relative to μ.The SlideLoss function is defined as:


$$\begin{array}{c} f(x)=\left\{ \;\;\;\;\;\;\;\;\;\;\begin{array}{c} \;\;\;\;\;\;\;\;\text{1},\\ e^{\text{1}-\mu }\\ {\;\;e}^{\text{1}-x}, \end{array},\right.\;\;\;\begin{array}{c} x\leq \mu -\text{0}.\text{1}\\ \mu <x<\mu -\text{0}.\text{1}\\ x\geq \mu \end{array} \end{array}$$ (17)

where x denotes the IoU value between the predicted and ground truth boxes, and μ represents the mean IoU of the current positive sample set.

When x approaches μ, the weight function f(x) increases, indicating that such samples are more likely to be “boundary hard samples” and thus receive greater loss contribution. Moreover, for samples with extremely high or low confidence, SlideLoss applies exponential gradient attenuation to prevent the model from biasing toward extreme samples during training.

**Fig 11:**
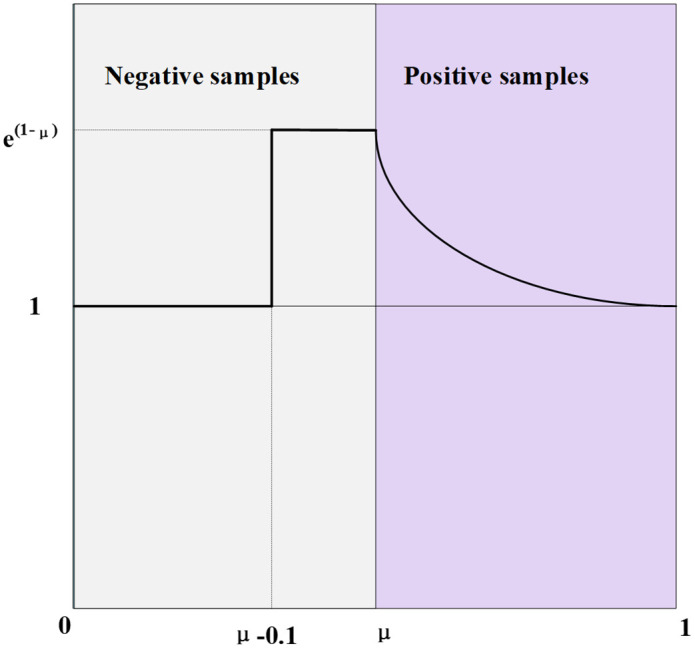
The schematic diagram of SlideLoss.

As illustrated in Fig 11, the weight curve peaks near the decision boundary, highlighting its ability to amplify supervision for ambiguous targets. This design allows the model to better distinguish subtle fractures and blurred anatomical structures, without the need for additional hyperparameters, offering both efficiency and robustness.

## 4. Experiments and results

### 4.1. Implementation details

To ensure experimental reproducibility and unbiased evaluation, the dataset is randomly partitioned into training, test, and validation sets using a 7:2:1 split, consisting of 14,228, 4,066, and 2,033 images, respectively. To enhance the model’s robustness and generalization capability, data augmentation is applied to the training set using the Albumentations library [45]. Various transformations, including random rotation, horizontal flipping, and brightness and contrast adjustment, are employed to simulate imaging variations and prevent overfitting. All experiments are conducted on a Windows 11 Pro 64-bit operating system equipped with an Intel(R) Core(TM) i7-14700HX processor, 16 GB of RAM, a 1 TB SSD, and an NVIDIA GeForce RTX 4070 Laptop GPU with 8 GB of VRAM. The implementation is based on Python 3.9 and PyTorch 2.6.0 under the CUDA 12.6 environment.

The model is trained using a batch size of 16 and an input image resolution of 640 × 640. The Stochastic Gradient Descent (SGD) optimizer is employed with a learning rate of 0.01. The training period of the model is set to 300 epochs, with one weight file saved per epoch, and the optimal weight file is selected as the final result.

### 4.2. Evaluation metrics

To comprehensively evaluate the performance of the proposed model on pediatric wrist abnormalities detection, four widely adopted metrics are utilized: Precision (P), Recall (R), F1 Score, and mean Average Precision (mAP). These metrics provide quantitative insight into both the classification confidence and localization accuracy of the model.

Precision measures the proportion of correctly predicted positive samples among all positive predictions and is formulated as:


$$\begin{array}{c} Precision=\frac{TP}{TP+FP} \end{array}$$ (18)

where TP denotes the number of true positive detections, and FP represents false positives.

Recall quantifies the ability of the model to retrieve all relevant positive instances and is defined as:


$$\begin{array}{c} Recall=\frac{TP}{TP+FN} \end{array}$$ (19)

where FN refers to the number of positive samples that are incorrectly cognition as negative samples.

The Average Precision (AP) is used to quantify a model’s detection performance by computing the area under the precision–recall (PR) curve. It is defined as:


$$\begin{array}{c} AP=\int_{\text{0}}^{\text{1}}P(R)dR \end{array}$$ (20)

where P(R) denotes the precision at a given recall level R. Based on this, the mean Average Precision (mAP) is computed as the mean of AP over all object categories, formulated as:


$$\begin{array}{c} mAP=\frac{\text{1}}{N}\sum_{i=\text{1}}^{N}AP_{i} \end{array}$$ (21)

where N is the total number of categories, and $AP_{i}\;$is the average precision of the i-th category. As a comprehensive evaluation metric, mAP reflects both the classification accuracy and localization precision of object detection models.

F1 Score is the harmonic mean of Precision and Recall, offering a balanced evaluation when both false positives and false negatives are of concern:


$$\begin{array}{c} F\text{1}=\text{2}\cdot \frac{Precision\cdot Recall}{precision+Recall} \end{array}$$ (22)

### 4.3. Ablation experiment results

To assess the contribution of each proposed component, we conducted stepwise ablation experiments from the baseline model (Form a) to the full MFSD-YOLO configuration (Form f), as summarized in Table 2 and Table 3. Each experiment incrementally introduces one or more modules to evaluate their effect on both overall performance metrics and per-category average precision (AP).

**Table 2:** Overall performance comparison of stepwise ablation experiments.

Form	CSP_PMSFA	FPSConv	C2BRA	RepGDFPN	SlideLoss	Pre(%)	Rec(%)	mAP_@0.5_(%)	mAP_@0.5:0.95_(%)
**a**	–	–	–	–	–	71.7	63.6	64.4	41.5
**b**	✓	–	–	–	–	71.7	63.3	66.6	41.6
**c**	✓	✓	–	–	–	70.9	67.6	67.9	42.9
**d**	✓	✓	✓	–	–	75.3	64.2	67.6	42.3
**e**	✓	✓	✓	✓	–	75.1	66.2	68.9	43.1
**f**	✓	✓	✓	✓	✓	77.3	64.0	69.7	43.0

**Table 3:** Per-category average precision (AP) results of ablation experiments.

Form	AP(%)						
	Boneanomaly	Bonelesion	Fracture	Metal	Periosteal reaction	Pronatorsign	Softtissue
**a**	26.6	34.4	93.9	96.4	68.8	66.7	28.6
**b**	36.0	39.2	94.3	97.6	70.7	66.6	29.1
**c**	45.1	38.1	94.5	97.9	71.4	65.9	31.2
**d**	40.2	38.8	94.5	97.8	72.1	66.3	32.0
**e**	44.2	56.6	94.5	98.0	70.0	65.3	23.6
**f**	34.8	60.5	94.3	97.8	71.2	67.6	32.2

The experimental results indicate that each proposed component contributed to enhancing the model’s performance to varying degrees. Starting from the baseline model (Form a), the introduction of the CSP_PMSFA module (Form b) improved mAP@0.5 from 64.4% to 66.6%, demonstrating that enhanced shallow feature extraction can positively impact overall detection accuracy. Incorporating the FPSConv module (Form c) yielded a more noticeable improvement in Recall (+3.5%) and increased mAP@0.5 to 67.9%, showing that expanding the receptive field significantly benefits target localization. The addition of the C2BRA attention mechanism (Form d) maintained relatively high Precision while slightly reducing Recall, resulting in an mAP@0.5 of 67.6%, suggesting that emphasizing boundary-related features stabilizes detection performance. With the integration of the RepGDFPN module (Form e), multi-scale feature fusion was strengthened, achieving a balanced improvement in both Precision and Recall, and raising mAP@0.5 to 68.9%. Finally, incorporating the SlideLoss function (Form f) further improved the model’s robustness in highly imbalanced class scenarios, achieving the highest mAP@0.5 of 69.7%.

From a category-level perspective, the Fracture class accounted for 66.6% of the dataset, with a sufficient number of samples, resulting in consistently high and stable average precision values above 94%. In contrast, other categories such as Boneanomaly (0.94%), Bonelesion (0.21%), Pronatorsign (2.78%), and Softtissue (2.16%) had relatively fewer and unevenly distributed samples, leading to lower and more variable AP values. The Foreignbody category (0.04%) had extremely limited samples, which resulted in many configurations failing to produce valid AP values. These findings highlight the significant class imbalance present in the dataset.

### 4.4. Visualization analysis

**Fig 12:**
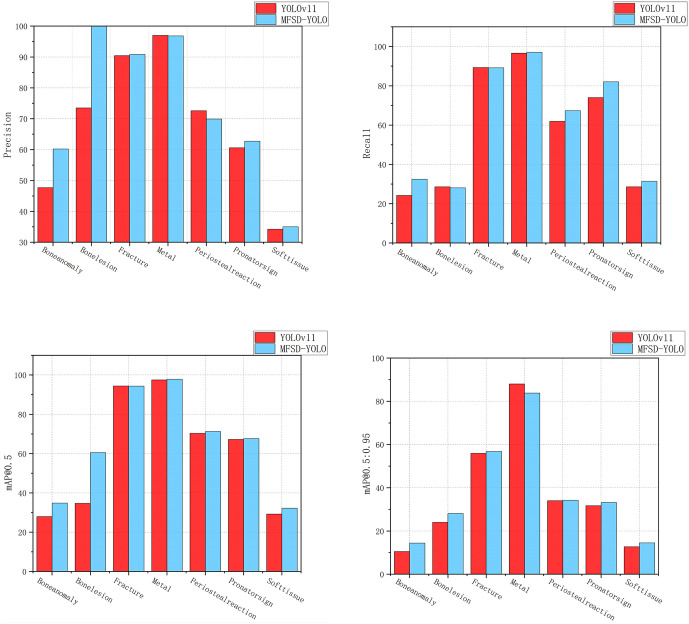
Comparison of training performance between MFSD-YOLO and YOLOv11s. (a) seven abnormalities training results for mAP@0.5, (b) seven abnormalities training results for mAP@0.5:0.95, (c) seven abnormalities t training results for precision, and (d) seven abnormalities training results for recall.

To evaluate the detection capabilities of the MFSD-YOLO model for 7 types of wrist abnormalities, both the original YOLOv11s model and the MFSD-YOLO model were trained using the same dataset and parameters. The performance of the two models was compared in terms of Precision, Recall, mAP@0.5, and mAP@0.5:0.95, as illustrated in Fig 12.

**Fig 13:**
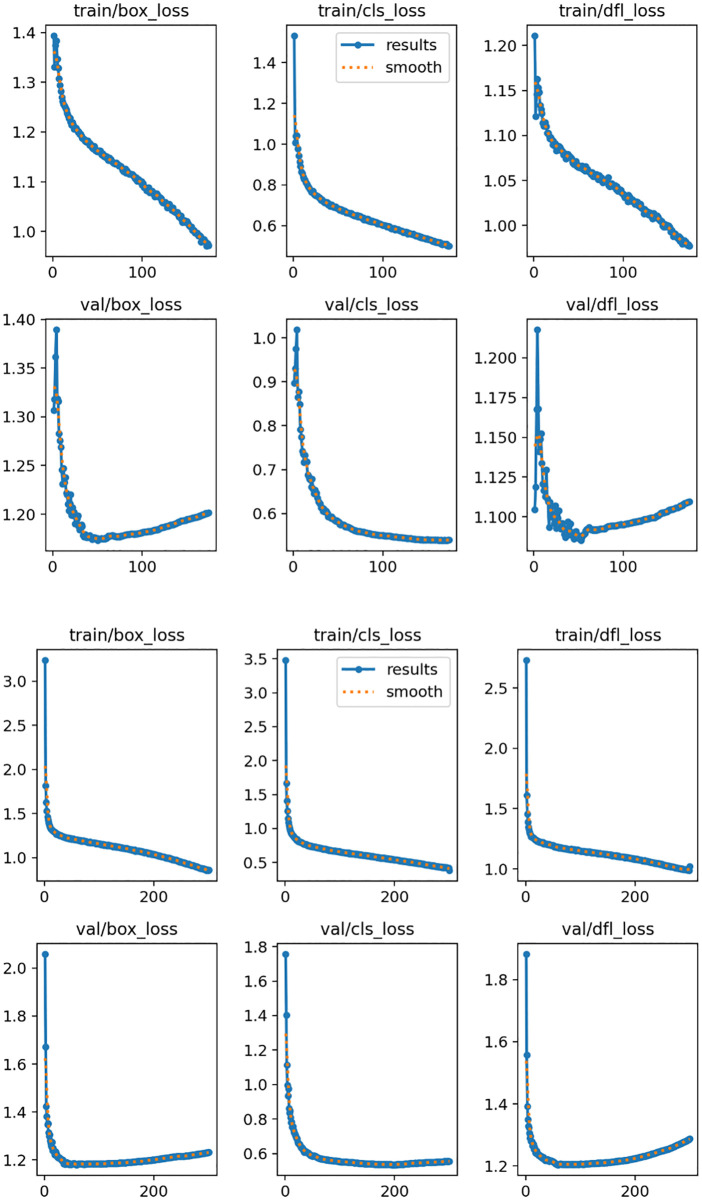
Training loss of YOLOv11and MFSD-YOLO. (a) Training loss of YOLOv11s, (b) Training loss of MFSD-YOLO.

Compared with YOLOv11s, MFSD-YOLO achieved notable improvements in Precision for detecting Bonelesion, Boneanomaly, and Pronatorsign. Slight enhancements in Recall were observed for Boneanomaly, Metal, Periosteal reaction, Pronatorsign, and Softtissue. For mAP@0.5, MFSD-YOLO delivered consistent improvements for Boneanomaly, Bonelesion, and Softtissue, indicating better localization accuracy at a relaxed IoU threshold. mAP@0.5:0.95 also showed slight improvements for Boneanomaly, Bonelesion, Fracture, Pronatorsign, and Softtissue, highlighting the robustness of the proposed model in detecting fine-grained and small-scale structures under stricter IoU constraints. The visualization of training results for YOLOv10s and RCA-YOLO is shown in Fig 13.

**Fig 14:**
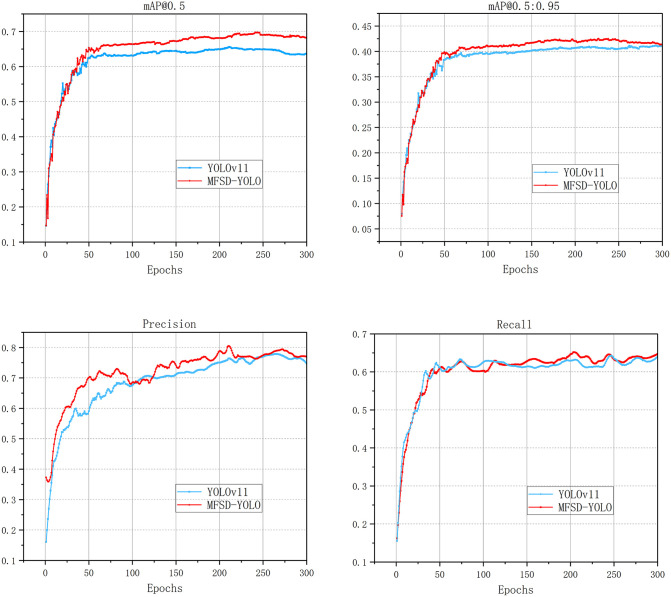
Comparison of training performance between MFSD-YOLO and YOLOv11. (a) training result of mAP@0.5, (b) training result of mAP@0.5:0.95, (c) training result of Precision, (d) training result of Recall.

As shown in the loss curves, both MFSD-YOLO and YOLOv11 exhibit a steady decline in training and validation losses during the initial training phase, indicating effective convergence. MFSD-YOLO demonstrates a slightly faster decrease in both losses within the first epochs, suggesting improved optimization stability. In the mid-to-late stages of training, the validation loss of MFSD-YOLO remains consistently lower and more stable than that of YOLOv11, with smaller fluctuations, reflecting better generalization to unseen data. In contrast, YOLOv11 shows relatively larger oscillations in the validation loss, particularly after reaching its minimum, which may indicate overfitting tendencies. Overall, MFSD-YOLO achieves lower final training and validation losses, implying more efficient learning and enhanced robustness. MFSD-YOLO outperformed YOLOv11s in terms of Precision, Recall, mAP, and mAP@0.5:0.95, highlighting its effectiveness in detecting wrist abnormalities. The results after 300 training epochs are presented in Fig 14. The proposed MFSD-YOLO algorithm consistently outperforms the baseline YOLOv11s model in wrist abnormality detection across all four evaluation metrics.

**Fig 15:**
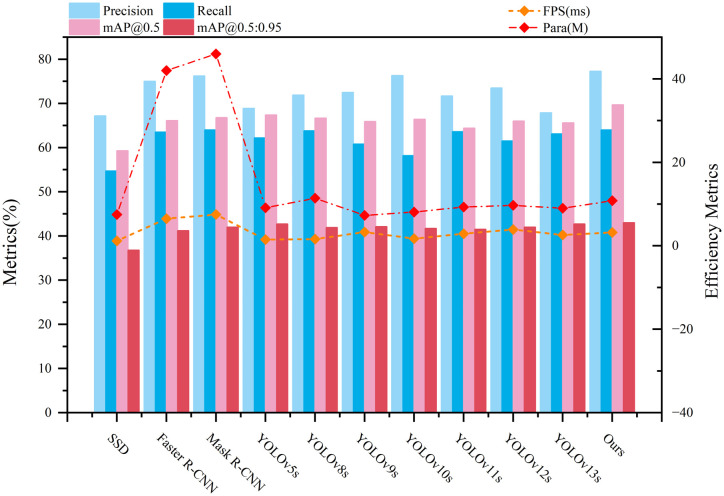
Comparison of Grad-CAM heatmaps.

To further assess the effectiveness of the proposed model in the context of pediatric wrist abnormalities detection, we employed the Grad-CAM visualization tool to generate class-discriminative heatmaps that highlight the regions of interest learned by each model. As shown in Fig 15, all models are evaluated under a high-resolution input setting (640 × 640). In these heatmaps, warmer colors (e.g., red or orange) denote higher attention weights, indicating areas that the model considers most salient for classification and localization. The distribution and concentration of these regions provide valuable insight into each model’s capacity to identify clinically relevant features within complex radiographic contexts.

The baseline YOLOv11s model exhibits only moderate activation intensity near fracture sites, however, its attention maps frequently present dispersed or misaligned saliency across non-discriminative anatomical structures. For instance, in image 2, although both a metallic implant and a fracture are present, YOLOv11s fails to identify the fracture and instead emphasizes the implant and surrounding textures. This results in a false negative for the fracture region and diminished clinical interpretability, reflecting insufficient feature discrimination when confronted with overlapping tissues or radiodense artifacts. In contrast, the proposed MFSD-YOLO consistently demonstrates sharper, more centralized activations that are well-aligned with expert-annotated fracture zones. In complex scenarios, such as the second sample involving dense metal structures, MFSD-YOLO effectively isolates the true fracture signal while suppressing irrelevant responses. Moreover, across all visualized cases, MFSD-YOLO yields higher detection confidence, indicating enhanced certainty and robustness in identifying fracture regions.

### 4.5. Anatomical error analysis

**Fig 16:**
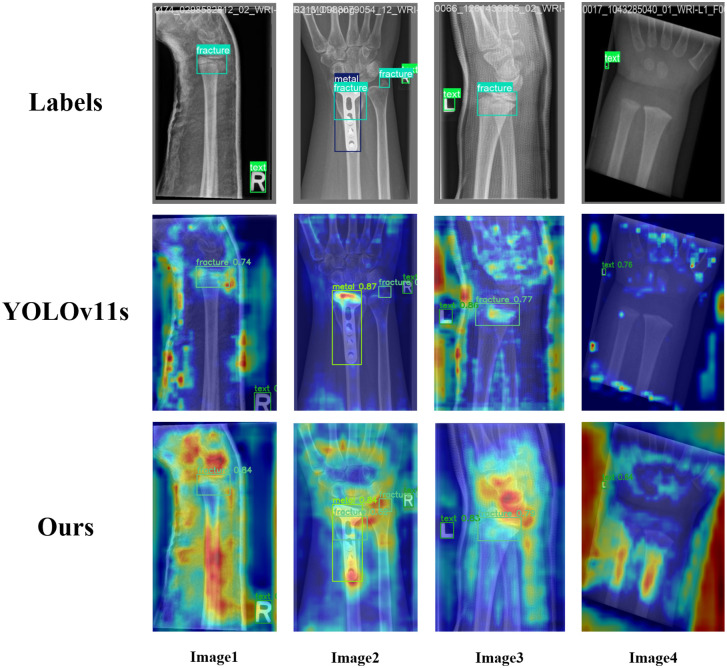
Visualization of typical misdetection cases of MFSD-YOLO.

To systematically evaluate MFSD-YOLO’s recognition deviations at the anatomical level, we conducted a case-by-case comparison between predicted results and ground truth (GT) labels in the test set. Errors were categorized into two types: false positives (FP) — instances where normal anatomical structures were incorrectly predicted as abnormalities; and false negatives (FN) — instances where actual abnormalities were missed. By examining the original radiographs, GT annotations, and model predictions, and referencing Grad-CAM heatmaps to assess activation regions, the anatomical structures associated with each misprediction were analyzed. Representative visualizations of misclassified cases are shown in Fig 16, highlighting differences between image appearance, GT labels, and model attention regions. Typical error types were further summarized by anatomical location and error nature (Table 4), revealing the predominant failure modes and potential anatomical limitations of MFSD-YOLO in pediatric wrist radiographs.

**Table 4:** Classification of major detection error types of MFSD-YOLO on pediatric wrist radiographs.

Error Category	Detection Type	Example	Error Manifestation
Small-scale/ low-contrast fracture missed	FN	Case 1	Missed subtle fracture
Incomplete detection when fracture coexists with secondary signs	FN	Case 2	Softtissue abnormality not detected
	FN	Case 3	Periosteal reaction missed; only fracture detected
	FN	Case 4	Multiple coexisting lesions (fracture, soft tissue, periosteal reaction) partially detected
Normal structures misclassified as fracture	FP	Case 5	Normal epiphyseal plate misidentified as fracture

The analysis indicates that the model’s failures mainly fall into three scenarios:

Missed small-scale/ low-contrast fractures (Case 1): In Case 1, the GT contains two fractures, but only the more prominent one is detected. This type of miss is primarily caused by the heterogeneous skeletal development in pediatric wrists: certain bones are partially ossified while others, such as the distal radial metaphysis, are nearly mature, resulting in scale differences from millimeters to centimeters and significant grayscale/density variations within a single image. Subtle fractures often manifest as very narrow linear density abnormalities with low contrast and poorly defined boundaries. When adjacent to the epiphyseal plate or obscured by overlapping bones, shallow-layer texture information can be weakened or lost during early convolutional feature extraction.

Incomplete detection when fractures coexist with secondary signs (Cases 2–4): When fractures coexist with soft tissue changes, periosteal reactions, or pronator quadratus signs, the model tends to prioritize high-contrast primary lesions (fractures) while underperforming or missing secondary signs. This occurs because secondary signs typically exhibit weak signals, irregular boundaries, or variable morphology, and may overlap with fractures in 2D projections. The severe class imbalance in the dataset exacerbates this issue: fracture samples (13,550 cases) overwhelmingly outnumber periosteal reaction (2,235), pronator sign (566), and soft tissue (439) samples, leading the network to focus on fracture discrimination while underrepresenting low-frequency, low-contrast secondary signs.

Normal anatomical structures or imaging artifacts misclassified as abnormalities (Case 5): Instances where GT labels show no pathology but the model falsely predicts a fracture. Normal pediatric wrist anatomical features, such as linear shadows of the epiphyseal plate, local density variations in volar or dorsal fat pads, and partially ossified cortical irregularities, can appear similar to fracture-like linear or band-shaped density abnormalities in single X-ray projections. Additionally, imaging angle, exposure settings, and overlapping soft tissue projections can generate artifacts resembling pathological disruptions.

From these cases and the dataset distribution, it is evident that MFSD-YOLO’s main failure modes have consistent anatomical and data-driven origins. First, the multi-scale anatomical heterogeneity of pediatric wrists (including differences in ossification, grayscale/texture similarity between epiphyseal plates and fracture lines, and bone–soft tissue superposition) inherently increases the difficulty of automated detection in single X-ray images. Second, the severe imbalance between fractures and secondary signs in GRAZPEDWRI-DX amplifies the model’s learning bias toward high-frequency fracture representations, weakening recognition of secondary signs and resulting in incomplete detection in multi-lesion scenarios. Overall, the observed errors are not random noise but can be explained through anatomical characteristics and dataset distribution logic.

### 4.6. Cross-Validation experiment

To comprehensively evaluate the stability and statistical significance of the MFSD-YOLO model, a five-fold cross-validation scheme was conducted on the GRAZPEDWRI-DX dataset. This design aims to reduce random biases arising from a single train/validation split, thereby providing a more robust and reliable assessment of model performance. Each fold underwent independent training and validation, and the resulting metrics included Precision, Recall, mAP@0.5, and mAP@0.5–0.95, along with their statistical distribution characteristics.

To assess the statistical significance of performance improvements, four statistical measures were calculated based on the results across folds: Mean, Standard Deviation (SD), 95% Confidence Interval (CI), and P-value. They are defined as follows:


$$\begin{array}{c} Mean=\frac{\text{1}}{n}\sum_{i=\text{1}}^{n}x_{i} \end{array}$$ (23)

$$\begin{array}{c} SD=\sqrt{\frac{\text{1}}{n-\text{1}}\sum_{i=\text{1}}^{n}{\left(x_{i}-Mean\right)}^{\text{2}}} \end{array}$$ (24)

$$\begin{array}{c} {CI}_{\text{9}\text{5}\%}=Mean\pm t_{\text{0}.\text{9}\text{7}\text{5},n-\text{1}}\times \frac{SD}{\sqrt{n}} \end{array}$$ (25)

$$\begin{array}{c} p=P\left(|t|\right)\geq \frac{\overset{―}{x_{\text{1}}}-\overset{―}{x_{\text{2}}}}{\sqrt{\frac{s_{\text{1}}^{\text{2}}}{n_{\text{1}}}+\frac{s_{\text{2}}^{\text{2}}}{n_{\text{2}}}}} \end{array}$$ (26)

where $x_{i}$denotes the performance value of the i-th fold, and $t_{\text{0}.\text{9}\text{7}\text{5},n-\text{1}}$is the critical value of the t-distribution with n−1degrees of freedom. The last equation represents a two-sample independent t-test used to determine whether the differences between the improved model (MFSD-YOLO) and the baseline model (YOLOv11) are statistically significant for each metric. A P-value less than 0.05 indicates that the observed performance improvement is statistically significant.

Table 5 summarizes the cross-validation statistics for YOLOv11 and MFSD-YOLO. Overall, MFSD-YOLO outperforms the baseline model across all four key metrics, with statistically significant improvements (p < 0.05). In particular, the mean mAP@0.5 is increased by approximately 8.7%, with the improvement being highly significant (p < 0.001), demonstrating that the proposed multi-scale fusion and structural enhancement modules reliably and reproducibly improve detection accuracy.

**Table 5:** Statistical summary of five-fold cross-validation results.

Metric	YOLOv11		MFSD-YOLO		P-value
	Mean±SD	95%CI		Mean±SD	95%CI
**Precision**	0.718 ± 0.0067	[0.709-0.726]	0.791 ± 0.0058	[0.784-0.798]	6.74 × 10 ⁻ ⁶
**Recall**	0.639 ± 0.0068	[0.630-0.647]	0.650 ± 0.0027	[0.646-0.653]	0.0119
**mAP@0.5**	0.642 ± 0.0063	[0.634-0.650]	0.698 ± 0.0038	[0.693-0.703]	1.49 × 10 ⁻ ⁵
**mAP@0.5-0.95**	0.412 ± 0.0068	[0.404-0.420]	0.432 ± 0.0027	[0.429-0.435]	0.00119

Furthermore, MFSD-YOLO exhibits smaller SD and narrower CI across the five folds compared to YOLOv11, indicating more stable performance under different data splits. In other words, the model not only achieves higher average accuracy but also demonstrates superior robustness and consistency. This suggests that the structural improvements in MFSD-YOLO—including CSP_PMSFA, FPSC, C2BRA, and RepGDFPN modules—effectively enhance the model’s feature representation of multi-scale and blurred structures in pediatric wrist radiographs, thereby reducing performance variability and improving overall generalization stability.

### 4.7. Comparative experiments

To comprehensively evaluate the performance of our proposed MFSD-YOLO model in pediatric wrist abnormalities detection, we conducted comparative experiments with several representative object detection frameworks, including SSD, Faster R-CNN, Mask R-CNN, YOLOv5s, YOLOv8s, YOLOv9s, YOLOv10s, YOLOv11s, YOLOv12s and YOLOv13s. All models were trained and tested under identical conditions on the GRAZPEDWRI-DX dataset to ensure fairness and consistency across evaluations. The experimental results are shown in Table 6.

**Table 6:** Comparison of detection performance of different detection models on the GRAZPEDWRI-DX dataset.

Model	Pre(%)	Rec(%)	mAP_@0.5_(%)	mAP_@0.5:0.95_(%)	FPS(ms)	Para(M)	Flops(GB)
**SSD**	67.2	54.7	59.3	36.8	1.2	7.5	17.6
**Faster R-CNN**	75.0	63.5	66.1	41.2	6.5	42.0	118.3
**Mask R-CNN**	76.2	64	66.8	42.0	7.5	46.0	134.0
**YOLOv5s**	68.9	62.2	67.4	42.7	1.5	9.1	24.1
**YOLOv8s**	71.9	63.8	66.7	41.9	1.6	11.4	28.7
**YOLOv9s**	72.5	60.8	65.9	42.1	3.3	7.3	27.4
**YOLOv10s**	76.3	58.2	66.4	41.7	1.7	8.1	24.8
**YOLOv11s**	71.7	63.6	64.4	41.5	2.9	9.3	21.9
**YOLOv12s**	73.5	61.5	66.0	42.0	3.9	9.7	24.7
**YOLOv13**	67.9	63.1	65.6	42.7	2.6	9.0	23.7
**Ours**	77.3	64.0	69.7	43.0	3.2	10.8	23.4

Classical detectors like SSD demonstrated relatively low performance, with mAP@0.5 of only 59.3% and recall as low as 54.7%, indicating insufficient sensitivity to detect fine-grained wrist abnormalities in pediatric radiographs. Although SSD offers extremely fast inference and a lightweight structure, its limited feature fusion and single-scale anchor design make it unsuitable for subtle lesion detection in complex anatomical backgrounds. Two-stage models such as Faster R-CNN and Mask R-CNN achieved improved accuracy over SSD, with mAP@0.5 values of 66.1% and 66.8%, respectively. The incorporation of region proposal networks (RPN) and segmentation heads in Mask R-CNN helps extract richer contextual features, which benefits fracture localization. However, these improvements come at the cost of computational overhead: both models exhibit inference latency exceeding 6.5 ms and model sizes above 40M, making them less practical for real-time deployment. Additionally, Faster R-CNN’s relatively low recall (63.5%) suggests suboptimal sensitivity in detecting subtle, discontinuous fracture patterns common in pediatric wrist X-rays.

Within the YOLO series, earlier variants such as YOLOv5s and YOLOv8s offered competitive performance in wrist abnormality detection while maintaining fast inference and relatively low complexity. YOLOv5s achieved a balanced precision and recall, while YOLOv8s maintained similar effectiveness but with slightly increased computational cost. However, their backbone and neck modules lacked dynamic adaptability to highly variable skeletal structures, limiting their capability to capture fracture-relevant semantics at multiple scales. Later versions such as YOLOv9s and YOLOv10s focused on structural modifications. YOLOv10s achieved the highest precision among all YOLO variants, indicating strong discriminative ability for clearly defined fracture boundaries. However, its recall dropped sharply to 58.2%, revealing that its detection sensitivity for faint or overlapping fractures remained inadequate. YOLOv9s, with its intermediate architecture, failed to balance detection quality, scoring a mAP@0.5 of 65.9%, suggesting neither architectural novelty nor parameter efficiency yielded significant advantage for this specific medical imaging task. The YOLOv11s and YOLOv12s models exhibited more consistent detection trends, benefiting from programmable gradient mechanisms and modular attention enhancements. Nonetheless, their performance plateaued at a mAP@0.5 of 64.4% and 66.0%, respectively, failing to effectively capture the multi-scale, low-contrast features critical to wrist abnormality detection. YOLOv13 achieved slight gains in recall (63.1%) and mAP@0.5:0.95 (42.7%), suggesting improved localization of subtle fractures. However, its precision dropped to 67.9%, reflecting reduced discrimination ability.

**Fig 17:**
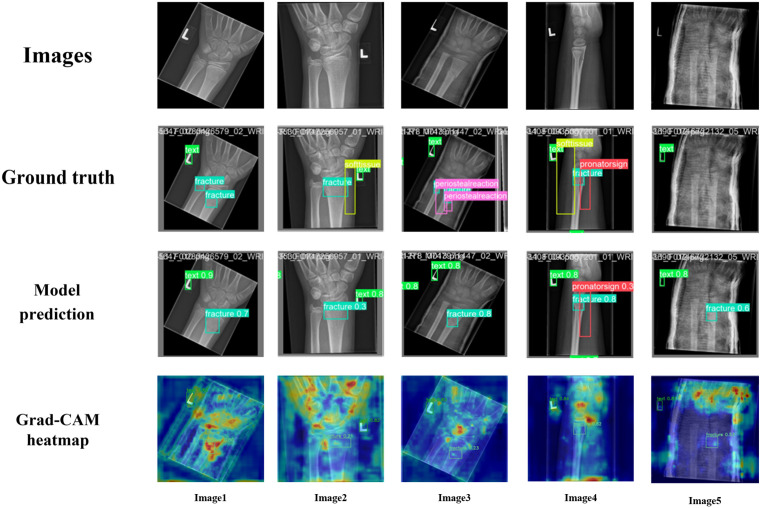
The comprehensive metrics of MFSD-YOLO and other models.

Our proposed MFSD-YOLO model outperformed all comparison methods in nearly every metric. It achieved the highest mAP@0.5 (69.7%) and mAP@0.5:0.95 (43.0%), reflecting robust detection performance across both easy and hard wrist abnormality instances. Importantly, the model maintained a competitive inference time of 3.2 ms and a modest parameter size of 10.8M, highlighting its feasibility for deployment in real-time clinical settings. In this study, we analyzed the comprehensive metrics of MFSD-YOLO and other models, as shown in Fig 17, by comparing the performance indexes of different models, it can be seen that the proposed method achieves the best balance in recognition performance, speed, and model size.

**Fig 18:**
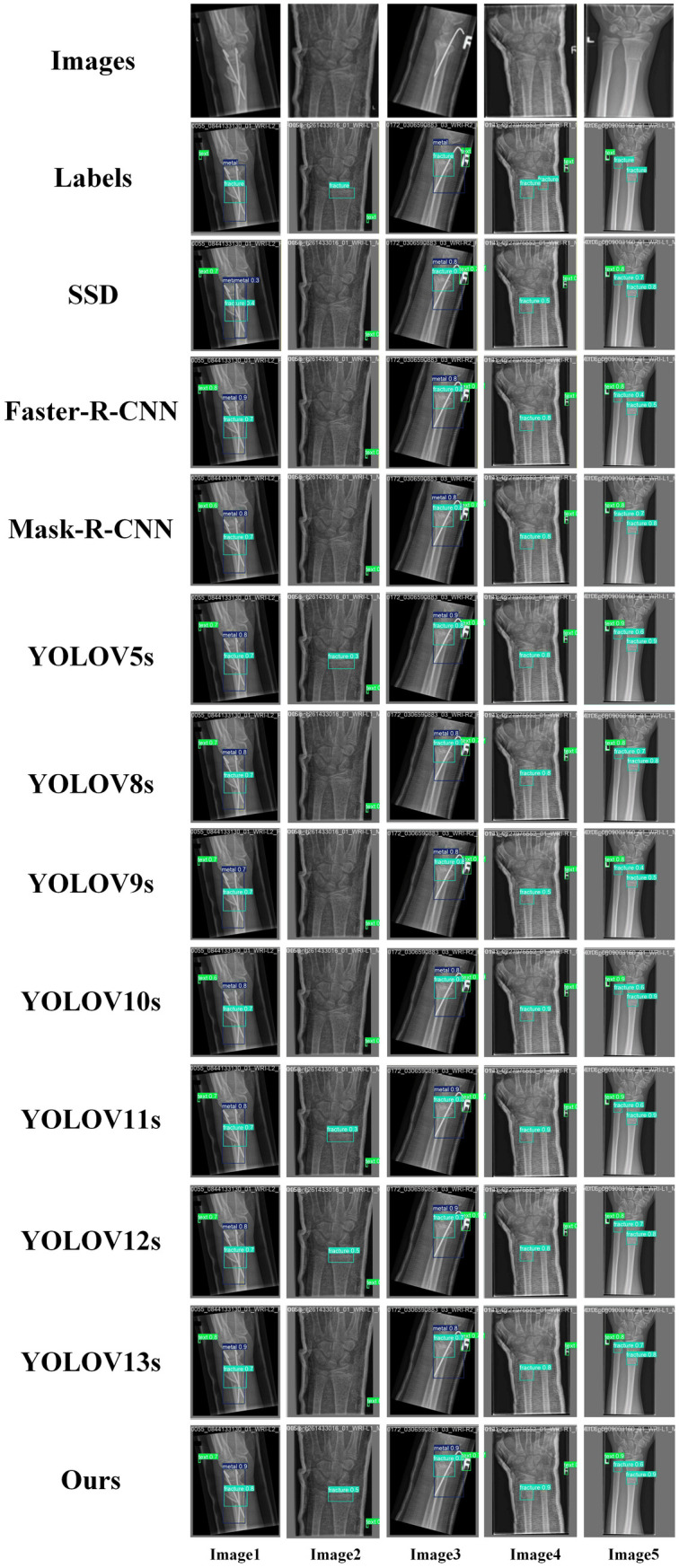
Detection results of different models.

To further validate the superiority of MFSD-YOLO in pediatric wrist radiograph detection, a multi-model visual comparison was conducted. As shown in Fig 18, the results demonstrate that MFSD-YOLO exhibits higher confidence and more complete detection performance across various complex imaging conditions.

In Images 1 and 3, all models successfully identified the fracture regions; however, MFSD-YOLO achieved significantly higher confidence scores with bounding boxes more closely aligned to the actual anatomical structures. This improvement is primarily attributed to the C2BRA module, whose channel–spatial collaborative attention mechanism effectively suppresses interference from non-structural high-intensity artifacts (such as metallic implants), maintaining stable focus on cortical bone boundaries under complex grayscale environments. Meanwhile, the CSP_PMSFA module enhances grayscale and texture preservation at shallow stages, enabling the model to distinguish subtle brightness variations at the bone–metal interface, thereby improving detection reliability under artifact occlusion.

In Image 2, MFSD-YOLO successfully detected a subtle fracture region that other models failed to recognize. The lesion exhibited low contrast and mild translucency, indicating that the progressive multi-scale convolution strategy of CSP_PMSFA plays a critical role in enhancing sensitivity to fine-grained cortical discontinuities. Furthermore, the FPSConv and RepGDFPN modules ensure semantic consistency during multi-scale feature fusion, preventing the loss of small-scale structures during upsampling. This design substantially increases the model’s sensitivity to early periosteal reactions or non-displaced fractures characterized by fine, low-contrast features.

In Image 5, although all models identified two fracture sites, MFSD-YOLO maintained the highest confidence, reflecting more stable overall feature representation and clearer response boundaries even in regions with weak grayscale contrast. Conversely, Image 4 presents a case of complex overlapping structures where all models missed one small fracture. This suggests that even with multi-layer structural optimization, challenges remain under extremely blurred or low signal-to-noise conditions. Future research will focus on integrating contextual modeling and anatomical prior learning to enhance robustness and interpretability in rare or complex pediatric wrist fracture cases.

Overall, these visual comparisons substantiate the advantages of MFSD-YOLO in terms of detection completeness, confidence calibration, and adaptability to complex bone morphology.

## 5. Discussion

### 5.1. Model performance and advantages

Accurately identifying fractures and related abnormalities in pediatric wrist radiographs remains a major challenge in medical image analysis. Existing studies often rely on fully convolutional networks or feature pyramid structures to enhance feature representation, but such approaches frequently suffer from fine-detail loss during encoding-decoding and semantic inconsistency across scales. Pediatric wrist images present additional difficulties: incompletely developed growth plates, gradual gray-level transitions between cortical and trabecular bone, and blurred margins of local fracture lines. These features result in lesions that are small, low-contrast, and morphologically irregular, making stable and high-precision detection difficult for conventional methods. To address these challenges, we propose MFSD-YOLO, a multi-scale detection model based on the YOLOv11 framework for automatic localization and identification of abnormalities in pediatric wrist radiographs.

Previous studies have introduced attention mechanisms or multi-level structural enhancements to improve detection performance. For instance, Ju et al. [18,20] and Chien et al. [23] incorporated attention modules into YOLO architectures to enhance feature focus on abnormal regions; Tariq et al. [22] combined ResNet with guided attention to improve global semantic modeling. While these methods achieve moderate improvements, most rely on simple module stacking and are not systematically designed to address the unique anatomical characteristics and clinical requirements of pediatric wrists. As a result, their performance in low-contrast, small-scale fractures or blurred boundaries remains unstable.

In contrast, MFSD-YOLO introduces targeted optimizations across four dimensions: structural representation, semantic focus, multi-scale alignment, and loss constraints. The CSP_PMSFA module introduces progressive multi-scale convolutions and partial convolutional branches within the CSP backbone, enhancing shallow texture and fine-grained feature extraction. This allows the model to better distinguish cortical bone, trabecular bone, and growth plate regions, even when fracture lines are faint or growth plates are incompletely developed. The C2BRA module combines channel and spatial attention to strengthen responses in clinically critical regions, such as lesions and growth plates, maintaining stability under low contrast and subtle fracture conditions. FPSConv and RepGDFPN ensure cross-scale feature consistency and spatial alignment via shared convolution and dynamic upsampling, enabling the model to capture both small distal fractures and the overall wrist structure while mitigating semantic drift caused by differences in bone size and projection angles. SlideLoss addresses class imbalance by suppressing bias toward dominant categories, maintaining sensitivity to rare lesions such as bone anomalies or foreign bodies, which enhances overall clinical reliability.

Compared to existing YOLO-based models, MFSD-YOLO demonstrates not only higher detection accuracy but also deeper integration of clinical image features into its architecture. Unlike lightweight models such as G-YOLO, MFSD-YOLO achieves precise anatomical-level feature modeling and cross-scale consistency while maintaining efficient inference (3.2 ms per image) and a manageable parameter count (10.8M). Overall, MFSD-YOLO represents a systematic optimization informed by medical imaging characteristics and clinical requirements rather than a simple module stacking, bridging generic visual detection with clinically meaningful semantic understanding.

### 5.2. Study limitations and future work

Despite encouraging results, this study has several limitations that must be acknowledged and indicate directions for future research. First, the model’s generalizability is limited by the single-center data source. All images used in this study were from the GRAZPEDWRI-DX dataset, originating from a single medical institution. This implies that the model may inadvertently have learned biases related to the specific equipment, imaging protocols, or patient population of that center. Performance may significantly decline when applied to images acquired under different settings or by other hospitals. Additionally, although SlideLoss mitigates the severe class imbalance in the dataset to some extent, detection performance for low-frequency categories such as “bone lesion” and “foreign body” remains unstable.

Second, there is a trade-off between model lightweight design and performance. While we prioritized efficiency, MFSD-YOLO (10.8M parameters) is not optimized for minimal computational resources. For example, as noted in the introduction, Ferdi et al. [45] designed a CAD system based on GhostNet and C3Ghost modules to minimize parameters for extremely resource-constrained devices. Our design prioritizes higher detection accuracy within acceptable computational cost (3.2 ms inference time). While reasonable in most clinical scenarios, this may limit real-time deployment on low-power mobile platforms.

Third, the model lacks integration with clinical information and intrinsic interpretability. MFSD-YOLO is purely image-based, without leveraging patient age, sex, or injury history. This absence of multimodal data limits diagnostic ceiling and renders the model a “black box,” making its decision process less transparent to clinicians—a key barrier to clinical adoption of deep learning models.

Moreover, the study did not specifically assess the model’s ability to distinguish normal anatomical variants (e.g., accessory ossification centers) from true fractures. While GRAZPEDWRI-DX provides annotations for fractures and major anatomical structures, it does not explicitly label these normal variants. The model may therefore generate false positives, potentially affecting clinical reliability. This limitation highlights the need for further evaluation of the model’s discriminatory ability for easily confusable anatomical structures.

Future work should expand to multi-center, multi-modal datasets, integrating X-ray, MRI, and clinical textual information to enhance generalizability across imaging conditions and patient populations. Techniques for attention visualization and feature saliency analysis can improve interpretability and support clinical decision-making. Lightweight optimization and edge deployment, including model pruning, structural re-parameterization, and knowledge distillation, are also important directions to reduce computational cost while maintaining accuracy. Additionally, prospective multi-center clinical validation is necessary to evaluate model stability and diagnostic benefits, along with dedicated assessment of normal anatomical variants to improve reliability in distinguishing physiological structures from pathological abnormalities.

### 5.3. Clinical implications and risks

From a clinical perspective, MFSD-YOLO holds promise as an effective computer-aided screening tool rather than a stand-alone diagnostic system. In high-volume medical centers where radiologists face substantial imaging workloads, the model could serve as a preliminary triage assistant, rapidly detecting and highlighting regions with potential fractures. By prioritizing suspicious cases, it may help radiologists allocate their time more efficiently and concentrate on complex or uncertain findings. In primary care settings or emergency departments where experienced pediatric radiologists are often unavailable, MFSD-YOLO could provide valuable decision-support information to non-specialist physicians. Such assistance may help improve the accuracy of initial assessments and reduce the likelihood of missed or delayed diagnoses that could otherwise lead to long-term complications such as epiphyseal injury or malunion.

However, it is crucial to stress that MFSD-YOLO, in its current form, is not a diagnostic tool. It should be regarded solely as a potential screening and decision-support system, whose outputs must always be interpreted and verified by qualified clinicians before reaching any clinical conclusion. The model’s application should remain limited to an auxiliary role within a controlled diagnostic workflow.

A primary barrier to clinical implementation lies in the model’s limited performance on rare entities. Although MFSD-YOLO demonstrates robust detection of common categories such as “fracture,” its accuracy and stability decline markedly when dealing with uncommon pathological findings, including “bone lesions” and “foreign bodies.” This inconsistency poses a substantial challenge to its safe deployment, as overreliance on the model could lead to false reassurance or missed diagnoses of rare but clinically significant abnormalities.

Moreover, generalizability remains another major concern. Since all data used in this study were collected from a single medical center, the model may have inadvertently learned center-specific biases related to imaging devices, acquisition protocols, or demographic patterns. Applying it directly to images acquired in other institutions with different equipment or settings could result in unpredictable performance degradation, increasing the likelihood of false-negative or false-positive results. Consequently, before any clinical integration, the model must undergo comprehensive, multi-center prospective validation on diverse datasets to ensure its robustness, reliability, and safety under real-world clinical conditions.

## 6. Conclusion

In this study, a novel pediatric wrist abnormalities detection model, termed MFSD-YOLO, is proposed to address the challenges of detecting small-scale, blurred-boundary, and fine-structured fracture patterns in pediatric radiographic images. The CSP_PMSFA module mitigates redundancy in intermediate features while enhancing shallow texture representation, whereas the FPSConv module broadens the receptive field and enriches contextual semantics through shared dilated convolutions. The C2BRA attention module, built upon the C2PSA framework, incorporates regional routing to focus computation on semantically relevant areas and applies local enhancement to refine fine-grained structures, thereby balancing modeling precision and inference speed for high-resolution medical images. The RepGDFPN neck improves bidirectional multi-scale feature fusion and reduces semantic loss during feature propagation, while the SlideLoss function adaptively enhances localization precision for subtle and ambiguous fracture regions. Experimental results on the GRAZPEDWRI-DX dataset validate the model’s effectiveness, achieving 69.7% mAP@0.5, 43.0% mAP@0.5:0.95, 77.3% precision, and 64.0% recall. Overall, these results confirm that MFSD-YOLO provides a robust and reliable framework for automated detection of pediatric wrist abnormalities, offering valuable assistance for clinical diagnosis and radiographic assessment.

## Supporting information

S1 File Training data. This file contains the training records of both the YOLO and MFSD-YOLO models for exactly 300 epochs.
(ZIP)

S2 File Cross-validation data. This file includes the detailed evaluation metrics from each round of cross-validation for both the YOLO and MFSD-YOLO models.
(ZIP)

S3 File Minimal data. This file provides a minimal representative subset of the dataset.
(ZIP)
