# Supplementary figures and images for: MFSD-YOLO: A multi-scale feature detection network for pediatric wrist abnormalities in radiographic images
Source: PLoS One. 2026 Feb 5;21(2):e0340408. doi: 10.1371/journal.pone.0340408 (PMC12875500; doi:10.1371/journal.pone.0340408)

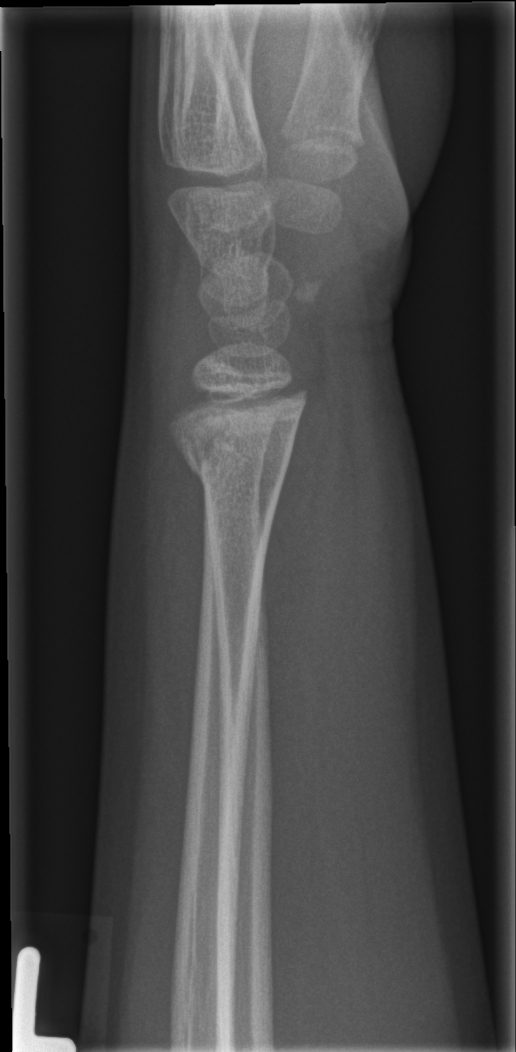

Supplement: S3 File — This file provides a minimal representative subset of the dataset. (ZIP) [file pone.0340408.s003.zip › minimal data/images/0015_0668695209_01_WRI-L2_F008.png]

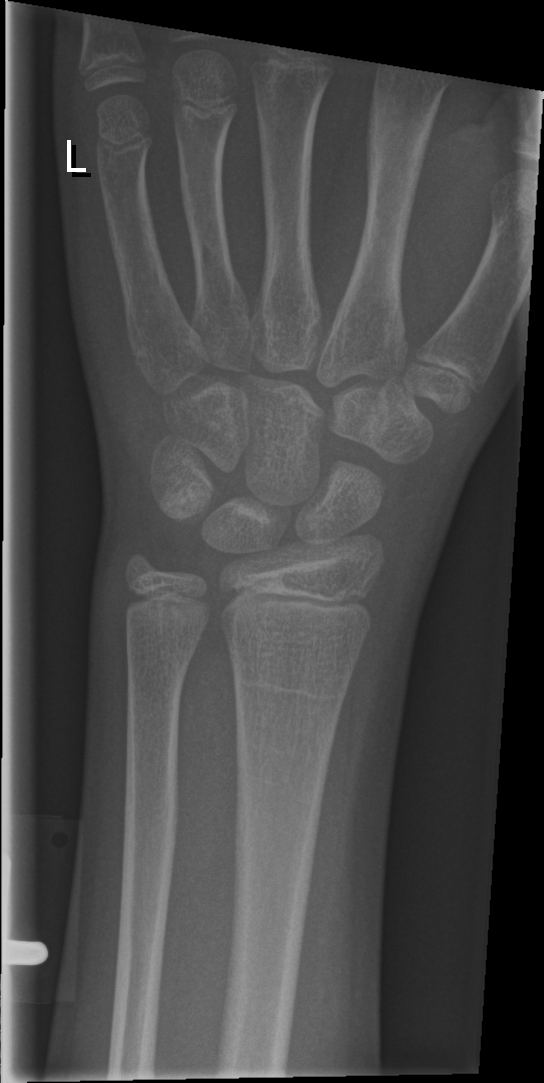

Supplement: S3 File — This file provides a minimal representative subset of the dataset. (ZIP) [file pone.0340408.s003.zip › minimal data/images/0016_0320600143_01_WRI-L1_F010.png]

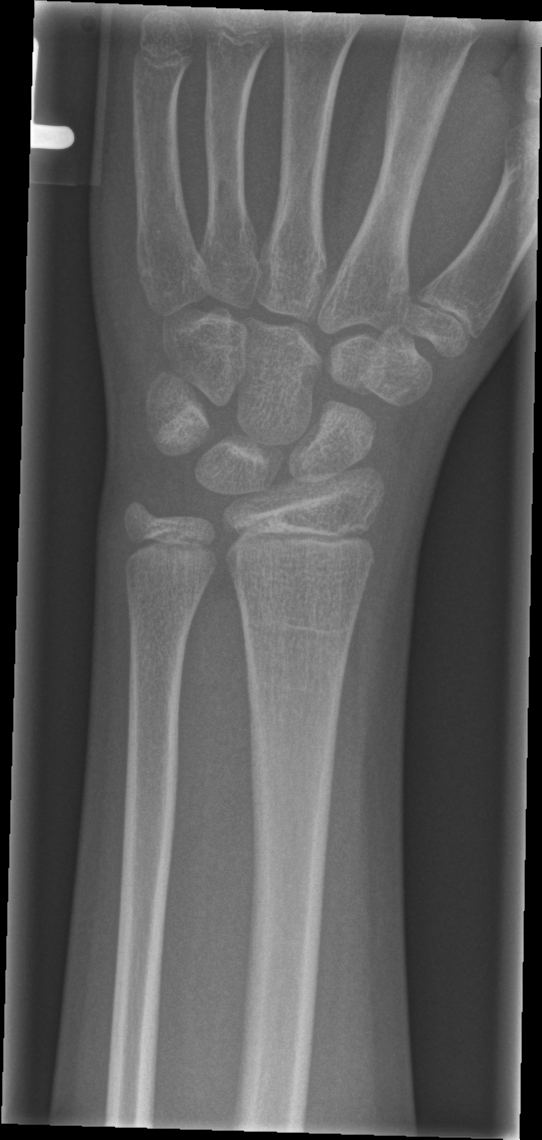

Supplement: S3 File — This file provides a minimal representative subset of the dataset. (ZIP) [file pone.0340408.s003.zip › minimal data/images/0016_0321123728_02_WRI-L1_F010.png]

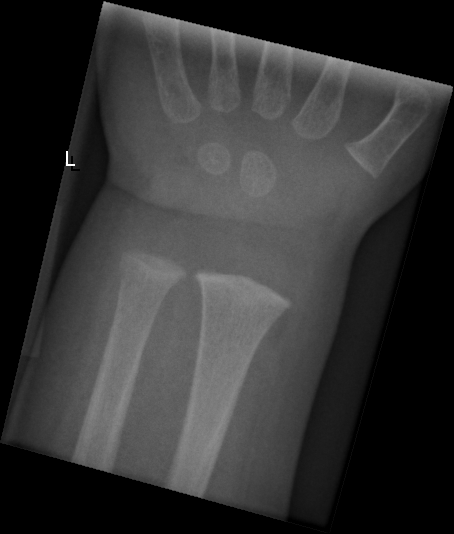

Supplement: S3 File — This file provides a minimal representative subset of the dataset. (ZIP) [file pone.0340408.s003.zip › minimal data/images/0017_1043285040_01_WRI-L1_F001.png]

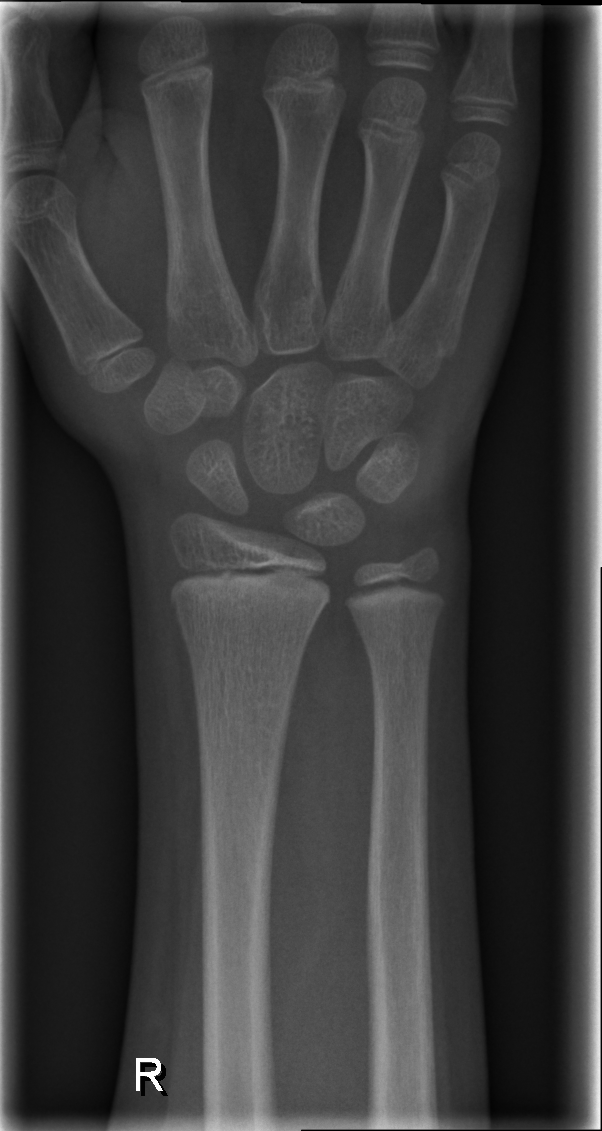

Supplement: S3 File — This file provides a minimal representative subset of the dataset. (ZIP) [file pone.0340408.s003.zip › minimal data/images/0019_0224740760_01_WRI-R1_M009.png]

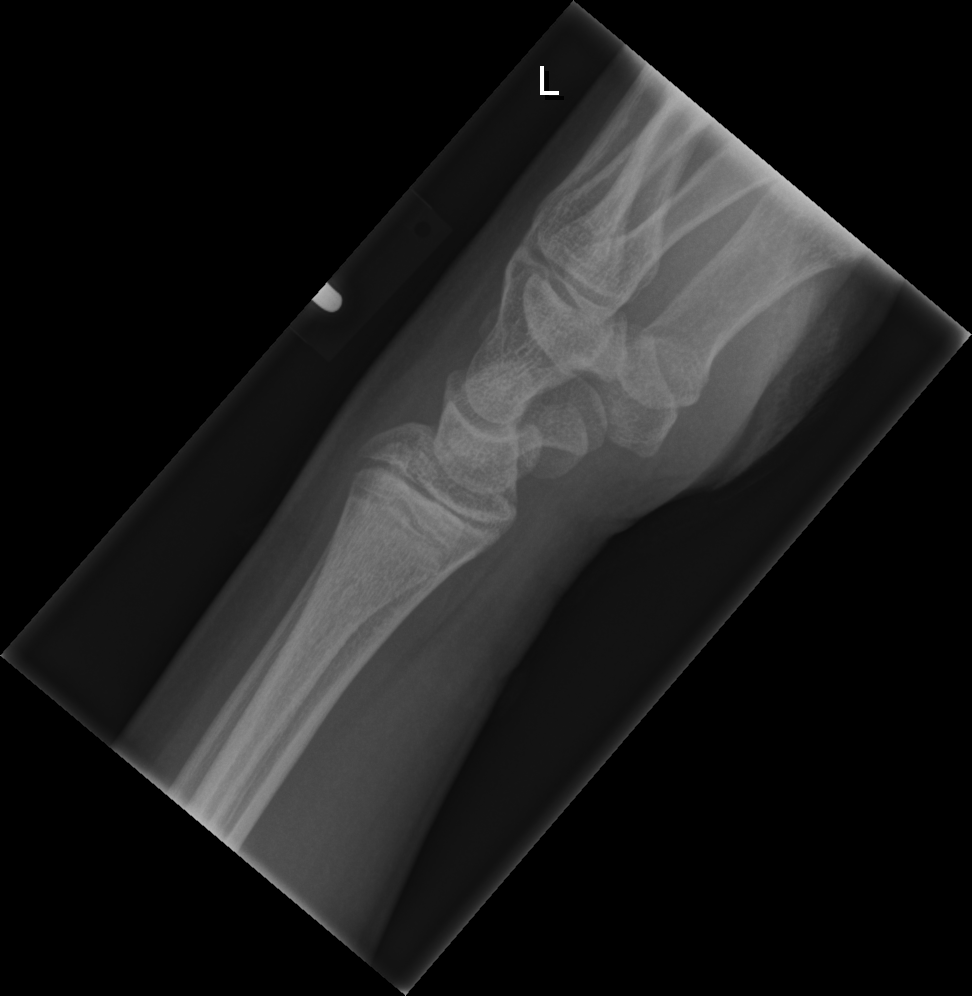

Supplement: S3 File — This file provides a minimal representative subset of the dataset. (ZIP) [file pone.0340408.s003.zip › minimal data/images/0020_0163123942_01_WRI-L2_M015.png]

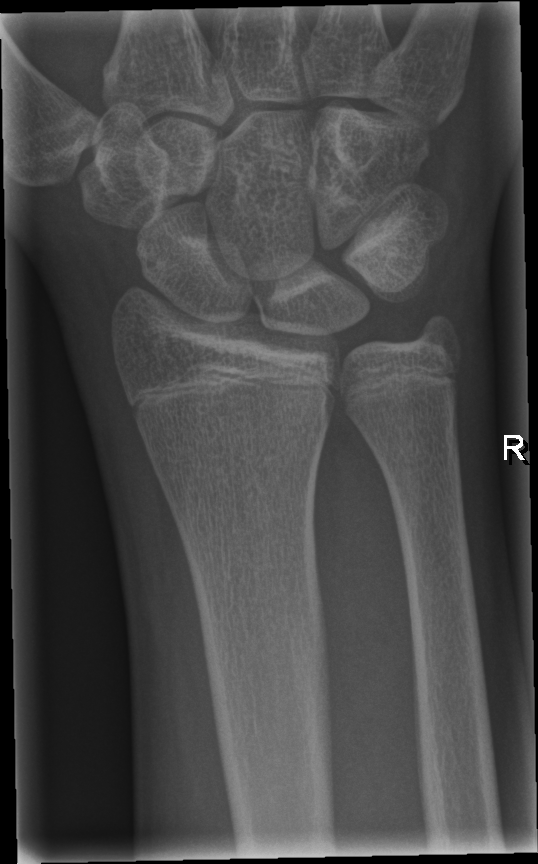

Supplement: S3 File — This file provides a minimal representative subset of the dataset. (ZIP) [file pone.0340408.s003.zip › minimal data/images/0021_1009239861_01_WRI-R1_M015.png]

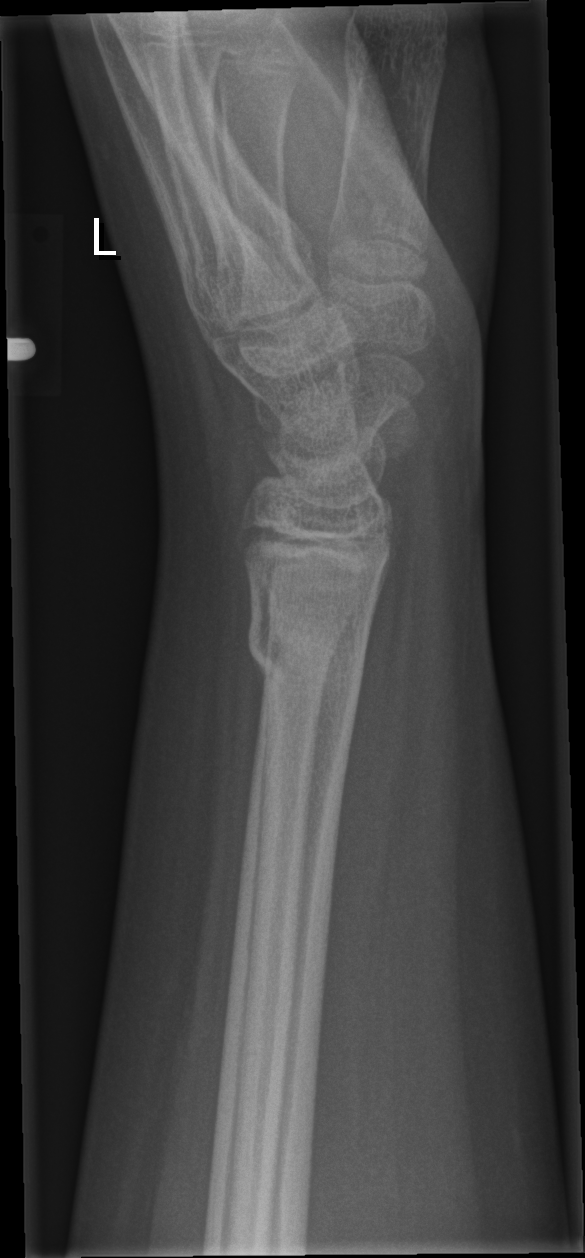

Supplement: S3 File — This file provides a minimal representative subset of the dataset. (ZIP) [file pone.0340408.s003.zip › minimal data/images/0022_0395494351_02_WRI-L2_M012.png]

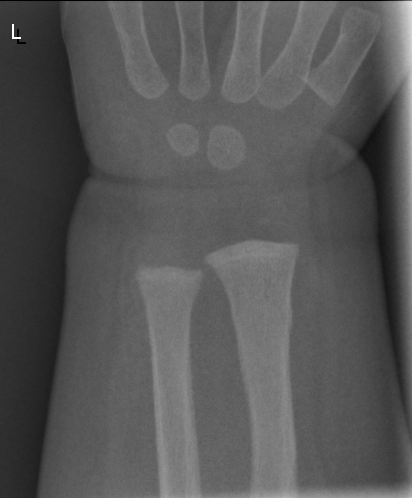

Supplement: S3 File — This file provides a minimal representative subset of the dataset. (ZIP) [file pone.0340408.s003.zip › minimal data/images/0025_0483842858_01_WRI-L1_F000.png]

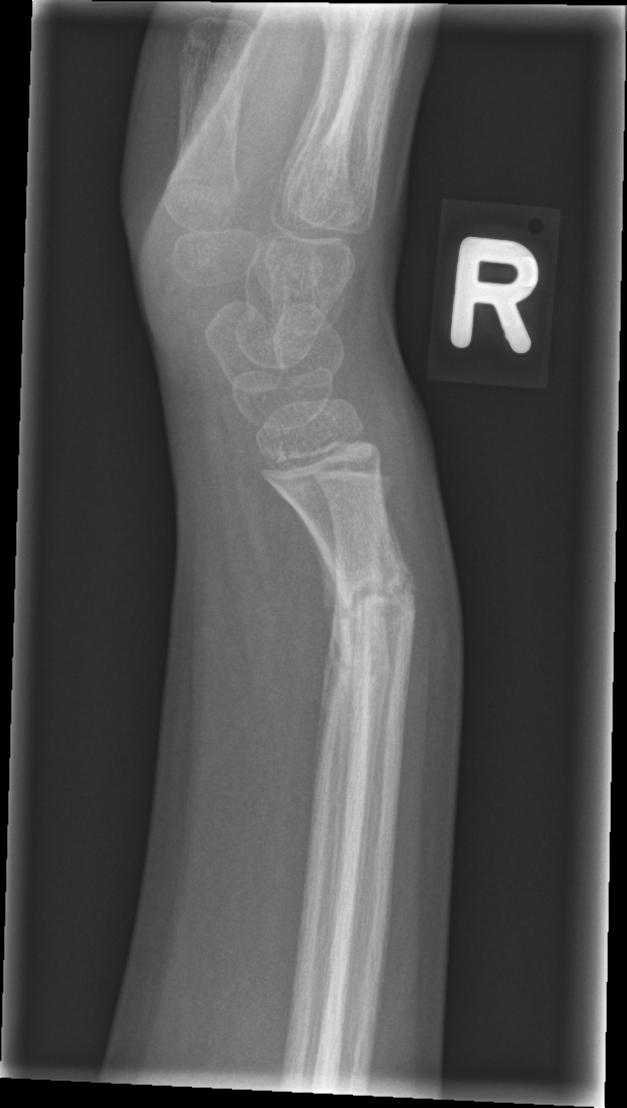

Supplement: S3 File — This file provides a minimal representative subset of the dataset. (ZIP) [file pone.0340408.s003.zip › minimal data/images/0028_0579540510_04_WRI-R2_M008.png]
